# CO_2_-sensitive tRNA modification associated with human mitochondrial disease

**DOI:** 10.1038/s41467-018-04250-4

**Published:** 2018-05-14

**Authors:** Huan Lin, Kenjyo Miyauchi, Tai Harada, Ryo Okita, Eri Takeshita, Hirofumi Komaki, Kaoru Fujioka, Hideki Yagasaki, Yu-ichi Goto, Kaori Yanaka, Shinichi Nakagawa, Yuriko Sakaguchi, Tsutomu Suzuki

**Affiliations:** 1 Department of Chemistry and Biotechnology, Graduate School of Engineering, University of Tokyo, 7-3-1 Hongo, Bunkyo-ku, Tokyo, 113–8656 Japan; 2 Department of Mental Retardation and Birth Defect Research, National Institute of Neuroscience, National Center of Neurology and Psychiatry, 4-1-1 Ogawahigashi, Kodaira, Tokyo, 187-8502 Japan; 3 Department of Child Neurology, National Center Hospital, National Center of Neurology and Psychiatry, 4-1-1 Ogawahigashi, Kodaira, Tokyo, 187-8502 Japan; 4 Department of Pediatrics, Interdisciplinary Graduate School of Medicine and Engineering, University of Yamanashi, 1110 Shimokato, Chuo, Yamanashi, Yamanashi, 409-3898 Japan; 5 Medical Genome Center, National Center of Neurology and Psychiatry, 4-1-1 Ogawahigashi, Kodaira, Tokyo, 187-8502 Japan; 6 RNA Biology Laboratory, RIKEN Advanced Research Institute, 2-1 Hirosawa, Wako, Saitama, 351-0198 Japan; 7 RNA Biology Laboratory, Faculty of Pharmaceutical Sciences, Hokkaido University, Kita 12-jo Nishi 6-chome, Kita-ku, Sapporo, 060-0812 Japan

## Abstract

It has been generally thought that tRNA modifications are stable and static, and their frequencies are rarely regulated. *N*^6^-threonylcarbamoyladenosine (t^6^A) occurs at position 37 of five mitochondrial (mt-)tRNA species. We show that YRDC and OSGEPL1 are responsible for t^6^A37 formation, utilizing *L*-threonine, ATP, and CO_2_/bicarbonate as substrates. *OSGEPL1*-knockout cells exhibit respiratory defects and reduced mitochondrial translation. We find low level of t^6^A37 in mutant mt-tRNA isolated from the MERRF-like patient’s cells, indicating that lack of t^6^A37 results in pathological consequences. Kinetic measurements of t^6^A37 formation reveal that the Km value of CO_2_/bicarbonate is extremely high (31 mM), suggesting that CO_2_/bicarbonate is a rate-limiting factor for t^6^A37 formation. Consistent with this, we observe a low frequency of t^6^A37 in mt-tRNAs isolated from human cells cultured without bicarbonate. These findings indicate that t^6^A37 is regulated by sensing intracellular CO_2_/bicarbonate concentration, implying that mitochondrial translation is modulated in a codon-specific manner under physiological conditions.

## Abstract (web summary)

Transfer RNA modifications play critical roles in protein synthesis. Here the authors reveal the t^6^A37 tRNA modification is dynamically regulated by sensing intracellular CO_2_ concentration in mitochondria, implying metabolic regulation of protein synthesis.

## Introduction

Post-transcriptional modifications play diverse roles in RNA structure and function and are associated with various biological processes and physiological events^1, 2^. To date, over 130 types of RNA modifications have been identified in various RNAs from all domains of life^3^. Transfer (t)RNAs contain multiple modifications involved in the stabilization of tRNA tertiary structure and precise decoding of genetic information^4, 5^. A wide variety of RNA modifications are present in the anticodon regions of tRNAs, where they make critical contributions to the accuracy of protein synthesis at various steps of translation, including aminoacylation, decoding, and translocation. In particular, tRNA modifications at the first position of the anticodon (wobble position) modulate codon–anticodon interactions at the ribosomal A-site. Another hot spot in this region is a hypermodification at position 37, 3′-adjacent to the anticodon. These modifications strengthen codon–anticodon pairings by enhancing base-stacking interactions, thereby helping to maintain the correct reading frame during decoding. Optimal rate of translation fine-tuned by tRNA modifications supports correct folding of proteins, thereby maintaining proteome integrity^6^.

The mitochondrion is a eukaryotic organelle that generates ATP through respiration. In mammalian mitochondria, 22 species of tRNAs encoded in mitochondrial (mt)DNA play essential roles in the translation of 13 essential subunits of the respiratory chain complexes required for oxidative phosphorylation^7^. These mt-tRNAs are modified post-transcriptionally by nuclear-encoded tRNA-modifying enzymes. Loss of tRNA modifications or tRNA-modifying enzymes is strongly associated with human disease caused by mitochondrial dysfunction^8^. In previous work, among all species of bovine mt-tRNAs, we identified 15 types of RNA modification at 118 positions^9^. Two types of taurine-containing modifications, 5-taurinomethyluridine (τm^5^U) and 5-taurinomethyl-2-thiouridine (τm^5^s^2^U), are mitochondria-specific modifications found at the wobble positions of mt-tRNAs responsible for two-codon sets ending in purines (NNR, R = A and G)^10^. We previously reported that τm^5^U34 and τm^5^s^2^U34 are not formed in mutant mt-tRNAs^Leu(UUR)^ isolated from patients with MELAS (mitochondrial encephalopathy, lactic acidosis, and stroke-like syndrome)^11^, or in mutant mt-tRNA^Lys^ isolated from patients with MERRF (myoclonus epilepsy with ragged-red fibers)^12^. Lack of these taurine modifications results in defective mitochondrial translation, leading to pathological consequences related to mitochondrial dysfunction^13, 14^. The pathogenic mutations in MELAS and MERRF are thought to act as negative determinants for the τm^5^U-modifying enzymes MTO1^15^ and GTPBP3^16^, and of the 2-thiouridylase MTU1^7, 17^. Consistent with this idea, whole-exome sequencing identified loss-of-function mutations in each of these three genes in patients with mitochondrial disorders^18–21^.

**Fig. 1:**
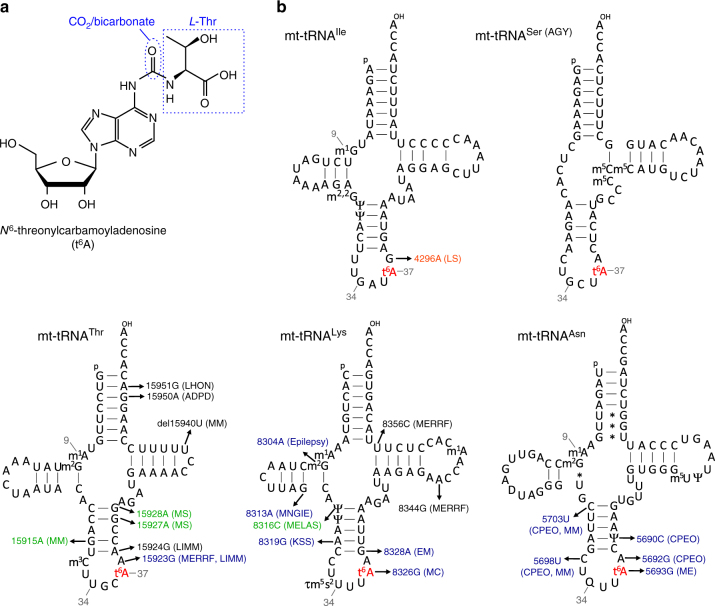
*N*^6^-threonylcarbamoyladenosine (t^6^A) in human mt-tRNAs. **a** Chemical structure of t^6^A. Carbonyl group derived from CO_2_/bicarbonate and Thr moiety are indicated. **b** Secondary structure and post-transcriptional modifications of five human mt-tRNAs bearing t^6^A37. Pathogenic point mutations deposited in MITOMAP^70^ are indicated in each tRNA. The color codes for the mutations are consistent with those in Fig. 5c, d. Abbreviations: 1-methylguanosine, m^1^G; *N*^2^,*N*^2^-dimethylguanosine, m^2,2^G; pseudouridine, Ψ; *N*^6^-threonylcarbamoyladenosine, t^6^A; 5-methylcytidine, m^5^C; 1-methyladenosine, m^1^A; *N*^2^-methylguanosine, m^2^G; 3-methylcytidine, m^3^C; 5-taurinomethyl-2-thiouridine, τm^5^s^2^U; queuosine, Q; 5-methyluridine, m^5^U; LHON Leber hereditary optic neuropathy; ADPD Alzheimer’s disease and Parkinson’s disease; MM mitochondrial myopathy; MS multiple sclerosis; LIMM lethal infantile mitochondrial myopathy; MERRF myoclonus epilepsy associated with ragged red fibers; EM encephalomyopathy; KSS Kearns Sayre syndrome; MELAS mitochondrial myopathy, encephalopathy, lactic acidosis, and stroke-like episodes; MNGIE mitochondrial neurogastro intestinal encephalomyopathy; CPEO chronic progressive external ophthalmoplegia; MC mitochondrial cytopathy; LS Leigh syndrome

*N*^6^-threonylcarbamoyladenosine (t^6^A) (Fig. 1a) and its derivatives are universally conserved modifications found at position 37 of tRNAs responsible for codons starting with A (ANN codons)^4, 22–26^. The bulky side chain of t^6^A extends its planar ring via intramolecular hydrogen bonds and stabilizes the anticodon loop structure by strengthening π–π stacking with adjacent bases and preventing base pairing with U33^27^. t^6^A37 enhances tRNA binding to the A-site codon, as well as its efficient translocation^28^, thereby helping to maintain the efficiency and accuracy of translation. t^6^A37 also plays critical roles in aminoacylation of tRNA^29^ and prevention of leaky scanning of initiation codons and read-through of stop codons^30^. In mammalian mitochondria, t^6^A37 is present in mt-tRNAs for Ser(AGY), Thr, Asn, Ile, and Lys (Fig. 1b)^9^. To date, however, the biogenesis and functional role of t^6^A37 in mammalian mt-tRNAs have not been elucidated.

The biogenesis of t^6^A has been well studied in bacterial systems. Formation of t^6^A on tRNA was successfully reconstituted in vitro using four essential enzymes, YrdC (TsaC), YgjD (TsaD), YeaZ (TsaB), and YjeE (TsaE), in the presence of the substrates *L*-threonine (Thr), ATP, and CO_2_/bicarbonate^31^. t^6^A is formed in two consecutive reactions^32^. First, YrdC employs Thr, CO_2_/bicarbonate, and ATP to synthesize threonylcarbamoyl-adenylate (TC-AMP), an active intermediate in t^6^A formation. Next, the three other enzymes (YgjD, YeaZ, and YjeE) catalyze nucleophilic attack of the *N*^6^-amino group of A37 on the carbonyl carbon of TC-AMP to synthesize t^6^A with release of AMP. YgjD is the catalytic subunit for t^6^A formation, and YeaZ and YjeE serve as accessory proteins that facilitate YgjD to recognize diverse tRNAs and synthesize t^6^A37. Structural studies revealed ATP-dependent assembly of the YgjD–YeaZ–YjeE complex^33^. The yeast cytoplasm contains the YrdC/TsaC homolog Sua5p and an ancient protein complex called KEOPS, which consists of five subunits, Kae1, Bud32, Cgi121, Pcc1, and Gon7. The YgjD homolog Kae1 serves a catalytic function, with the assistance of the other four subunits, to regulate formation of t^6^A in cytoplasmic tRNAs^34–36^. Homozygous pathogenic mutation related to global developmental delay was found in *OSGEP* gene, a human homolog of *Kae1*^37^. In addition, recessive mutations were found in four components of KEOPS complex in 32 families of Galloway–Mowat syndrome^38^, indicating that hypomodification of t^6^A in cytoplasmic tRNAs results in severe inherited disorders. On the other hand, in yeast mitochondria, the minimum enzyme system required for synthesis of t^6^A37 in mt-tRNAs consists of only two components: Sua5p and Qri7p^39, 40^. One isoform of Sua5p is localized to mitochondria and provides TC-AMP. Qri7p, a mitochondrial homolog of YgjD/Kae1, catalyzes t^6^A37 formation utilizing TC-AMP generated by mitochondrial Sua5p. *Arabidopsis thaliana* GCP1, a homolog of YgjD/Kae1, is localized to mitochondria, and is required for embryonic development in plants^41^.

YRDC and OSGEPL1 are human homologs of YrdC/Sua5 and YgjD/Qri7, respectively, and are predicted to be involved in t^6^A37 formation in mt-tRNAs^9^. In vivo function of these homologs were confirmed by complementation of *Escherichia coli yrdC* and *ygjD* mutants^37^. In this study, we demonstrated that YRDC and OSGEPL1 are responsible for t^6^A37 formation in five species of mt-tRNAs. OSGEPL1 knockout cells exhibited respiratory defects and reduced mitochondrial translation, suggesting that t^6^A37 plays a critical role in this process. We successfully reconstituted t^6^A37 formation in mt-tRNAs with recombinant YRDC and OSGEPL1 in the presence of Thr, ATP, and bicarbonate. Kinetic studies revealed that bicarbonate concentration is the rate-limiting factor for t^6^A37 formation in mitochondria. We observed hypomodification of t^6^A37 in mt-tRNAs isolated from human cells cultured in the absence of CO_2_ and bicarbonate, indicating that t^6^A37 formation in mt-tRNAs is sensitive to intracellular bicarbonate concentration. We also identified several pathogenic mutations in mt-tRNA genes that impaired t^6^A37 formation and confirmed that the level of t^6^A37 was reduced in mt-tRNA^Thr^ bearing the A15923G mutation isolated from MERRF fibroblasts and myoblasts, indicating that the absence of t^6^A37 has pathological consequences.

## Results

### Involvement of YRDC in t^6^A37 formation in mitochondria

**Fig. 2:**
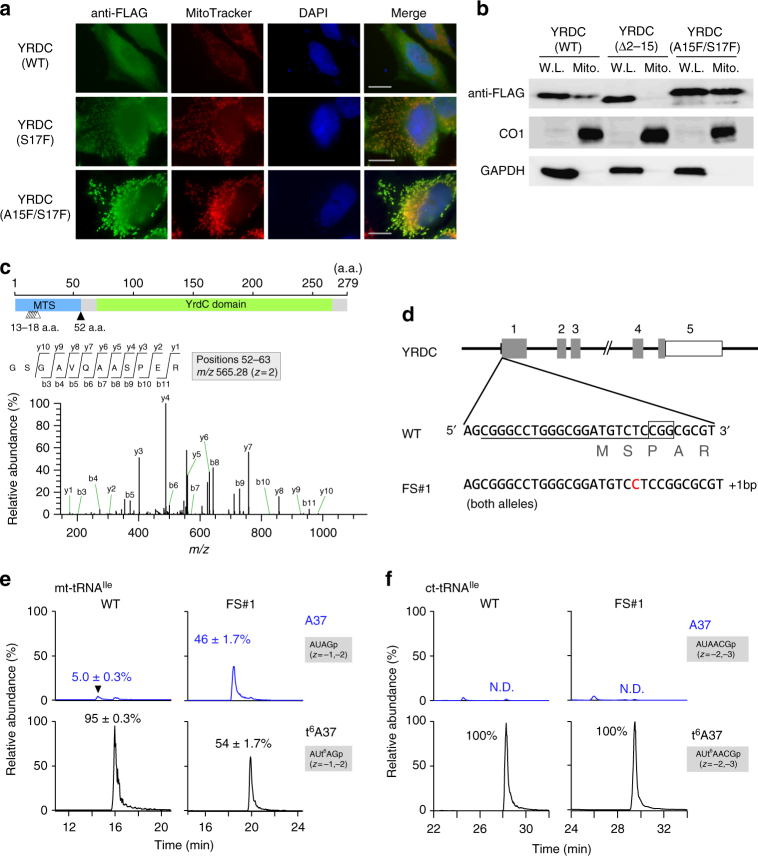
YRDC is responsible for t^6^A37 formation in mt-tRNAs. **a** Subcellular localization of wild-type (WT) and mutant YRDC (S17F, A15F/S17F) in HeLa cells immunostained with an anti-FLAG antibody (Green). Nuclei and mitochondria were stained with DAPI (blue) and MitoTracker (Red), respectively. All images were superimposed to generate the merged panel. Scale bars: 20 μm. **b** Mitochondrial localization of YRDC. Whole-cell lysates (W.L.) and mitochondrial fractions (Mito.) from HEK293T cells transfected with WT, variant with N-terminal truncation (a.a. Δ2–15), and mutant (A15F/S17F) YRDC constructs were subjected to western blotting with anti-FLAG antibody to detect YRDC variants, anti-CO1 (mitochondrial marker), and anti-GAPDH (cytoplasmic marker). Uncut gel images are provided in Supplementary Fig. 15. **c** Determination of the cleavage site in the MTS of YRDC. Schematic depiction of YRDC with a predicted MTS (blue) and a conserved region homologous to that of *E. coli* YrdC (green). Multiple cleavage sites in the long and the short isoforms of YRDC expressed in HeLa cells are indicated by white and black arrowheads, respectively. The CID spectrum represents the sequence of the N-terminal tryptic peptide of the short isoform with the cleavage site at position 52. The precursor ion for CID is *m/z* 565.28. Product ions are assigned on the peptide sequence. **d** Top: schematic of human *YRDC* gene and the site of insertion introduced with the CRISPR–Cas9 system. Shaded boxes indicate coding regions; open boxes indicate untranslated regions of exons; lines indicate introns. Inset: exon 1 of WT YRDC. The target sequence of the single guide RNA (sgRNA) is underlined; the protospacer adjacent motif (PAM) sequence is outlined. Bottom: sequence of the frameshift cell line (FS#1); the inserted C is indicated in red. **e** Extracted ion chromatograms (XICs) generated by integration of multiply-charged negative ions of the A37-containing fragments of human mt-tRNA^Ile^ with A37 (top) or t^6^A37 (bottom) (Supplementary Table 1) isolated from WT (left) and FS#1 (right) cell lines. t^6^A frequencies (%) are described as mean values ± s.d. of technical triplicate. **f** XICs generated by integration of multiply-charged negative ions of A37-containing fragments of human ct-tRNA^Ile(IAU)^ with A37 (top) or t^6^A37 (bottom) (Supplementary Table 1) isolated from WT (left) and FS#1 (right) cell lines

YRDC (Uniprot: Q86U90) (Supplementary Fig. 1a), a human homolog of YrdC/Sua5, was supposed to catalyze TC-AMP formation. To examine the subcellular localization of human YRDC, we transiently expressed C-terminally FLAG-tagged YRDC in HeLa cells and detected the tagged protein by immunostaining. As shown in Fig. 2a, YRDC was widely diffused throughout the cell, but tended to be more strongly localized to the cytoplasm. As determined by WoLF PSORT^42^, a tool for predicting protein localization, YRDC has an N-terminal mitochondrial targeting sequence (MTS) (Supplementary Fig. 1a and Fig. 2c), implying mitochondrial localization. To confirm this prediction, we isolated the mitochondrial fraction from HEK293T cells expressing YRDC-FLAG and performed western blotting alongside whole-cell lysate as a control. The purity of the mitochondrial fraction was confirmed by the absence of GAPDH signal (a cytoplasmic marker) and a strong CO1 signal (cytochrome c oxidase subunit I) (Fig. 2b). We clearly detected YRDC-FLAG in the mitochondrial fraction (Fig. 2b). In cells expressing an YRDC-FLAG variant with an N-terminal truncation (Δ2–15), very little signal was detected in the mitochondrial fraction, although it was detected in whole-cell lysate (Fig. 2b). We then constructed two YRDC-FLAG variants with MTS mutations, S17F and A15F/S17F, both of which improve PSORT scores for mitochondrial localization. For both variants, clear signals were observed in mitochondrial regions (Fig. 2a), indicating that the MTS mutations promoted mitochondrial localization of YRDC-FLAG. Consistent with this, western blotting (Fig. 2b) showed a strong signal in the mitochondrial fraction corresponding to the A15F/S17F variant. Because YRDC has a weak MTS, a large fraction of YRDC localizes in the cytoplasm and participates in t^6^A37 formation in cytoplasmic tRNAs, whereas a smaller fraction of YRDC is imported to mitochondria where it performs the same function for mitochondrial tRNAs. The MTS is frequently cleaved by mitochondrial processing protease after import^43^. To determine the cleavage sites in the MTS, we immunoprecipitated YRDC-FLAG and subjected the precipitated protein to mass-spectrometric analysis. We detected seven tryptic peptides derived from the N-terminus of YRDC (Fig. 2c and Supplementary Fig. 2a, b), indicating that multiple cleavages took place in mitochondria. Each peptide was sequenced by collision-induced dissociation (CID) to identify its N-terminus (Fig. 2c and Supplementary Fig. 2c), revealing six long isoforms with cleavage sites at positions 13–18 and one short isoform with the cleavage site at position 52 (Supplementary Fig. 1a, 2a). The existence of these truncated forms of YRDC supports our conclusion that a subset of YRDC is imported into mitochondria.

To confirm that YRDC is involved in mitochondrial t^6^A formation, we tried to knock out *YRDC* in HEK293T cells using the CRISPR/Cas9 system. However, we failed to obtain a null allele, indicating that *YRDC* is essential for cell viability. Next, we re-designed the single guide RNA (sgRNA) to target the N-terminus of YRDC, with the hope of obtaining cell lines harboring a mutation in the MTS (Fig. 2d). In this manner, we obtained a cell line, FS#1, in which one nucleotide was inserted at the second codon of YRDC in both alleles (Fig. 2d). We then used reciprocal circulating chromatography to isolate both mitochondrial and cytoplasmic (ct-)tRNAs^Ile^ from FS#1^44^ and analyzed the status of t^6^A37 by capillary LC-nano-ESI-mass spectrometry (RNA-MS)^45^. As shown in Fig. 2e, we detected RNase T_1_-digested fragments with t^6^A37 or A37 for both tRNAs (Supplementary Table 1) isolated from WT and FS#1 cells. To estimate ESI detection efficiencies of RNA fragments with and without t^6^A modification, we performed calibration curves for RNase T_1_-digested fragments of human mt-tRNA^Ile^ with varying t^6^A frequency (Supplementary Fig. 3). The regression line with a slope of 1 exhibited *r*^2^ = 0.9913, suggesting that t^6^A does not affect largely on ESI detection efficiency of RNA fragments. Thus, we can relatively quantify t^6^A frequency from their intensities of mass chromatograms.

In mt-tRNA^Ile^, the t^6^A37 frequency in WT cells (95%) was clearly reduced to 54% in FS#1 cells; consistent with this, a large fraction (46%) of hypomodified mt-tRNA^Ile^ was detected in FS#1 cells. By contrast, no reduction of t^6^A37 frequency was observed in ct-tRNA^Ile^ from FS#1 (Fig. 2f), indicating that the +1 frameshift mutation partially impaired mitochondrial localization of YRDC. Given that cytoplasmic t^6^A was not impaired in FS#1 cells, we hypothesize that Met at position 10 of YRDC (Supplementary Fig. 1a) functions as an alternative initiation site for production of cytoplasmic YRDC in this cell line. Otherwise, if mitochondrial localization of YRDC is completely impaired by the +1 frameshift mutation, TC-AMP produced by cytoplasmic YRDC might be imported to mitochondria by penetrating mitochondrial membranes, and used for mitochondrial t^6^A37 formation.

### OSGEPL1 is involved in t^6^A37 formation in mitochondria

**Fig. 3:**
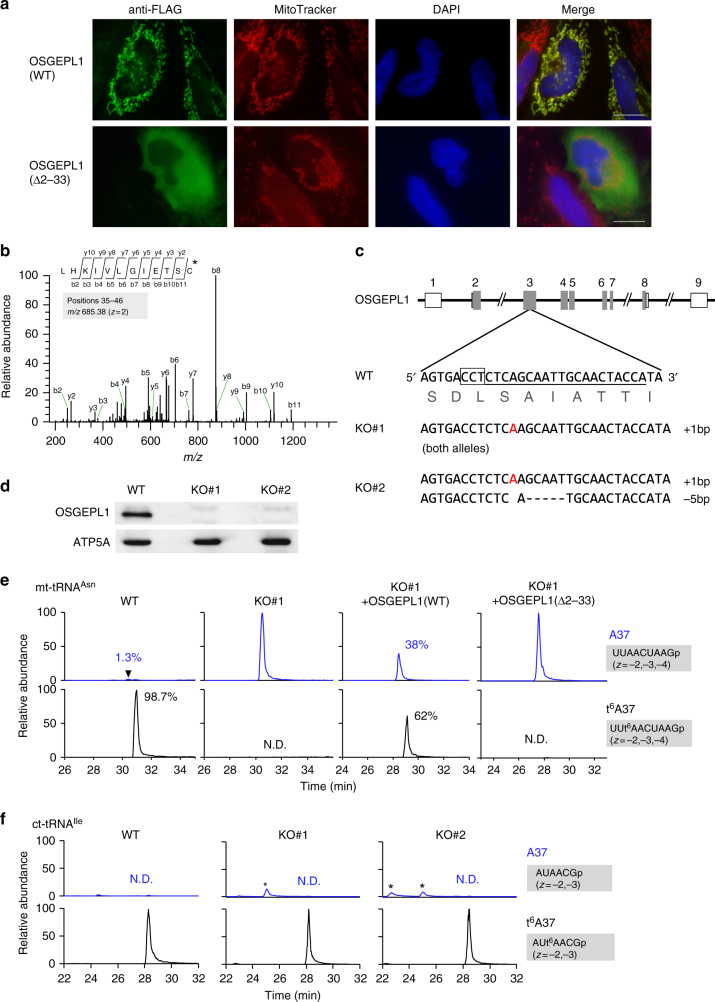
OSGEPL1 is responsible for t^6^A37 formation in mt-tRNAs. **a** Subcellular localization of wild-type (WT) and N-terminal truncated (a.a. Δ2–33) OSGEPL1 in HeLa cells immunostained with an anti-FLAG antibody (Green). Nuclei and mitochondria were stained with DAPI (blue) and MitoTracker (Red), respectively. All images were superimposed to generate the merged panel. Scale bars: 20 μm. **b** Determination of the cleavage site in the MTS in OSGEPL1. The CID spectrum represents a sequence of N-terminal peptide of OSGEPL1 starting from position 35. The precursor ion for CID is *m/z* 685.38. Product ions are assigned on the peptide sequence. C* stands for alkylated cysteine residue. **c** Top: schematic of human *OSGEPL1* gene and sites of mutations introduced by the CRISPR–Cas9 system. Shaded boxes indicate coding regions; open boxes indicate untranslated regions of exons; lines indicate introns. Inset: exon 3 of WT OSGEPL1. The target sequence of the single guide RNA (sgRNA) is underlined; the protospacer adjacent motif (PAM) sequence is outlined. Bottom: sequences of KO#1 and KO#2 cell lines. The inserted sequence is indicated in red and deleted sequences are indicated by dashed lines. **d** Confirmation of KO lines by western blotting of endogenous OSGEPL1 and the mitochondrial marker ATP5A (as a control). Uncut gel images are provided in Supplementary Fig. 15. **e** XICs generated by integration of multiply-charged negative ions of the A37-containing fragments of human mt-tRNA^Asn^ bearing A37 (top) or t^6^A37 (bottom) (Supplementary Table 1) isolated from WT, KO#1, and KO#1 rescued by plasmid-encoded OSGEPL1 (WT and a.a. Δ2–33). N.D., not detected. Intensity fractions (%) of modified or unmodified fragments are indicated. **f** XICs generated by integration of multiply-charged negative ions of the A37-containing fragments of human ct-tRNA^Ile(IAU)^ bearing A37 (top) and t^6^A37 (bottom) (Supplementary Table 1) isolated from WT, KO#1, and KO#2

OSGEPL1 (Uniprot: Q9H4B0) (Supplementary Fig. 1b), a human homolog of YgjD/Qri7, is predicted to be involved in t^6^A37 formation in mitochondria^9^. To explore this possibility, we examined the subcellular localization of C-terminally FLAG-tagged OSGEPL1 transiently expressed in HeLa cells. As shown in Fig. 3a, OSGEPL1 was predominantly localized in mitochondria. Consistent with this, iPSORT analysis^46^ predicted the existence of an MTS in the N-terminal region (positions 1–33) of OSGEPL1 (Supplementary Fig. 1b). The OSGEPL1 variant with the MTS truncation (Δ2–33) did not translocate to mitochondria, but was instead diffused throughout the cytoplasm (Fig. 3a). To determine the cleavage site in the MTS, we immunoprecipitated OSGEPL1-FLAG expressed in HEK293T cells and subjected the precipitated protein to MS. We detected an N-terminal peptide starting at position 35 (Fig. 3b and Supplementary Fig. 1b), indicating that a 34-a.a. N-terminal peptide was removed in mitochondria. This finding is consistent with the previous report that endogenous OSGEPL1 was detected in mitochondrial fraction of HeLa cells by cross-reactivity of *A. thaliana* anti-GCP1 antibody^41^.

Next, we knocked out *OSGEPL1* in HEK293T cells using the CRISPR/Cas9 system and obtained two cell lines (KO#1 and KO#2) with frameshift mutations in both alleles (Fig. 3c). No endogenous OSGEPL1 was detected in either cell line by western blotting (Fig. 3d). Next, we isolated five species of mt-tRNAs containing t^6^A37, along with ct-tRNA^Ile^ as a control, from WT cells and the two KO lines, digested each tRNA with RNase T_1_, and subjected the digested products to RNA-MS. We detected RNase T_1_-digested fragments containing t^6^A37 (Supplementary Table 1) from all tRNA species isolated from WT cells. Judging from the ratio of modified (t^6^A37) versus unmodified (A37) fragments, more than 62% (62–97%) of tRNAs contained the t^6^A37 modification (Fig. 3e and Supplementary Fig. 4). In all five species of mt-tRNAs isolated from *OSGEPL1*-KO cells, t^6^A37 was completely absent and converted to unmodified A37 (Fig. 3e and Supplementary Fig. 4). On the other hand, as expected based on the involvement of the KEOPS complex in cytoplasmic t^6^A formation, the t^6^A37 frequency was not altered in ct-tRNA^Ile^ isolated from the KO cells (Fig. 3f). These results demonstrated that OSGEPL1 is exclusively involved in t^6^A37 formation in mitochondria.

During RNA-MS analysis of mt-tRNA^Thr^ (Fig. 1b) isolated from the *OSGEPL1*-KO cells, we detected hypomodification of 3-methylcytidine (m^3^C) at position 32 (Supplementary Table 1). The high m^3^C frequency (94%) in WT cells was reduced to 67% in KO#1 and to 64% in KO#2 (Supplementary Fig. 5a), suggesting that t^6^A37 is required for efficient m^3^C32 formation.

When *OSGEPL1*-KO cells were rescued by plasmid-encoded *OSGEPL1*, t^6^A37 levels were partially restored (Fig. 3e). By contrast, t^6^A37 was not rescued by MTS-truncated *OSGEPL1* (a.a. Δ2–33) (Fig. 3e), suggesting that mitochondrial localization mediated by the MTS is essential for t^6^A37 formation in mt-tRNAs.

### Loss of OSGEPL1 causes mitochondrial dysfunction

**Fig. 4:**
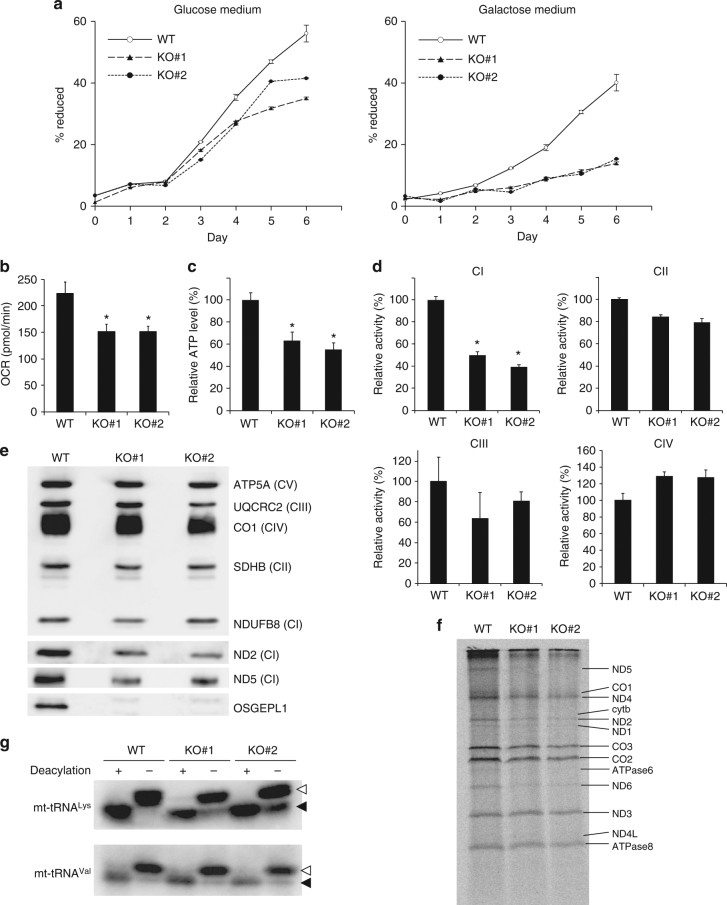
Mitochondrial dysfunction in *OSGEPL1* KO cells. **a** Growth curves for WT HEK293T, *OSGEPL1* KO#1, and *OSGEPL1* KO#2 cells cultured in the presence of glucose (left) or galactose (right) as the primary carbon source. Mean values ± s.e.m. of four independent cultures are plotted. **b** Oxygen consumption rates of WT, *OSGEPL1* KO#1, and *OSGEPL1* KO#2 cells measured using an XFp extracellular flux analyzer. Mean values ± s.d. of biological triplicates are compared. *P < 0.05, Student’s *t*-test. **c** Steady-state levels of ATP in WT, *OSGEPL1* KO#1, and *OSGEPL1* KO#2 cells. Mean values ± s.e.m. of three independent experiments are compared. *P < 0.05, Student’s *t*-test. **d** Relative activities of respiratory complexes I, II, III, and IV (CI–IV) in WT, *OSGEPL1* KO#1, and KO#2 cells. Mean values ± s.e.m. of three independent experiments are compared. *P < 0.05, Student’s *t*-test. **e** Steady-state levels of subunit proteins in respiratory chain complexes. Mitochondrial fractions of WT, *OSGEPL1* KO#1, and KO#2 cells resolved by SDS-PAGE were analyzed by western blotting with the indicated antibodies. A gel stained with Coomassie brilliant blue (CBB) is shown in Supplementary Fig. 6a. Uncut gel images are provided in Supplementary Fig. 15. **f** Pulse labeling of mitochondrial protein synthesis. WT, *OSGEPL1* KO#1, and KO#2 cells were labeled with [^35^S] methionine and [^35^S] cysteine after cytoplasmic protein synthesis was halted with emetine. Whole-cell lysates were resolved by Tricine-SDS-PAGE and stained with CBB as a loading control (Supplementary Fig. 6b). The dried gel was exposed to an imaging plate and visualized on a fluorimager. Assignment of mitochondrial proteins is indicated. Uncut gel images are provided in Supplementary Fig. 15. **g** In vivo aminoacylation levels of mt-tRNA^Lys^ and mt-tRNA^Val^ in WT, *OSGEPL1* KO#1, and *OSGEPL1* KO#2 cells. Crude aminoacyl-tRNAs extracted under acidic conditions were treated with (+) or without (−) mild alkali for deacylation and subjected to acid urea-PAGE and northern blotting. White and black arrowheads indicate aminoacyl-tRNA and deacyl-tRNA, respectively. Aminoacylation level of each condition was calculated from the band intensities. Lysylation levels of mt-tRNAs^Lys^ from WT, KO#1, and KO#2 are 98.4%, 89.9%, and 83.9%, respectively. Valylation levels of mt-tRNAs^Val^ from WT, KO#1, and KO#2 are 90.1%, 90.3%, and 89.1%, respectively. Uncut gel images are provided in Supplementary Fig. 15

Next, we assessed mitochondrial activity of *OSGEPL1*-KO cells by monitoring cell growth in medium containing galactose, a non-fermentable sugar, as the primary carbon source^47^. *OSGEPL1*-KO cells grew slightly slower than WT cells in glucose medium (Fig. 4a), but exhibited a severe growth defect in galactose medium (Fig. 4a), indicating mitochondrial dysfunction in *OSGEPL1*-KO cells. Measurement of respiratory activity and ATP levels in *OSGEPL1*-KO cells revealed that the oxygen consumption rate (OCR) (Fig. 4b) and ATP level (Fig. 4c) in KO cells were significantly lower than those in WT cells. To explore this phenomenon in greater details, we biochemically compared respiratory complex activities between *OSGEPL1*-KO and WT cells (Fig. 4d). We observed a clear reduction in Complex I, whereas no significant change was observed in other complexes. Consistent with this, the steady-state levels of ND2 and ND5, both of which are subunits of Complex I were markedly reduced in *OSGEPL1*-KO cells (Fig. 4e and Supplementary Fig. 6a). We also observed a slight reduction in the level of NDUFB8, a nuclear-encoded complex I subunit, indicating that assembly of Complex I was impaired in *OSGEPL1*-KO cells. To examine mitochondrial translational activity, we carried out a pulse-labeling experiment (Fig. 4f and Supplementary Fig. 6b). Specifically, we treated *OSGEPL1*-KO and WT cells with emetine to inhibit cytoplasmic translation and then added [^35^S] methionine and [^35^S] cysteine to the medium to specifically label mitochondrial translational products. Mitochondrial protein synthesis was clearly lower in *OSGEPL1*-KO cells than in WT cells (Fig. 4f). In particular, the levels of subunits of Complex I, including ND1, ND2, ND4, ND5, and ND6, were markedly reduced. Given that t^6^A37 has pleiotropic functions in protein synthesis, this result can be attributed to the hypomodification of mt-tRNAs in *OSGEPL1*-KO cells.

t^6^A37 is required for efficient aminoacylation in bacterial tRNAs^29^. Accordingly, we next measured the aminoacylation levels in mt-tRNA^Lys^ by acid-urea northern blotting (Fig. 4g). In WT cells, mt-tRNA^Lys^ was fully lysylated, whereas we clearly detected deacylated mt-tRNA^Lys^ in *OSGEPL1*-KO cells. Based on band intensities, the aminoacylation levels of mt-tRNA^Lys^ were 98.4% in WT, 89.9% in KO#1, and 83.9% in KO#2. As an important control, we observed no alteration of aminoacyl-mt-tRNA^Val^, which has no t^6^A37, in *OSGEPL1*-KO cells. This finding demonstrated that t^6^A37 is required for efficient aminoacylation of mt-tRNA^Lys^ in human mitochondria. To determine the steady-state level of the five mt-tRNAs in *OSGEPL1*-KO cells, we carried out northern blotting, but found no significant alteration in the levels of any of these tRNAs (Supplementary Fig. 7), indicating that lack of t^6^A37 has little effect on tRNA stability.

### In vitro reconstitution of mitochondrial t^6^A37 formation

In general, most tRNAs from all domains of life have highly conserved intramolecular tertiary interactions, including the D-loop/T-loop ‘kissing’ interaction that promotes folding into the L-shaped tertiary structure. However, most mammalian mt-tRNAs lack such D-loop/T-loop interactions and instead adopt non-canonical cloverleaf structures^7^. In fact, the sizes and sequences of the D/T-loops in t^6^A-containing mt-tRNAs vary considerably (Fig. 1b). In particular, mt-tRNA^Ser(AGY)^ lacks the entire D-arm.

**Fig. 5:**
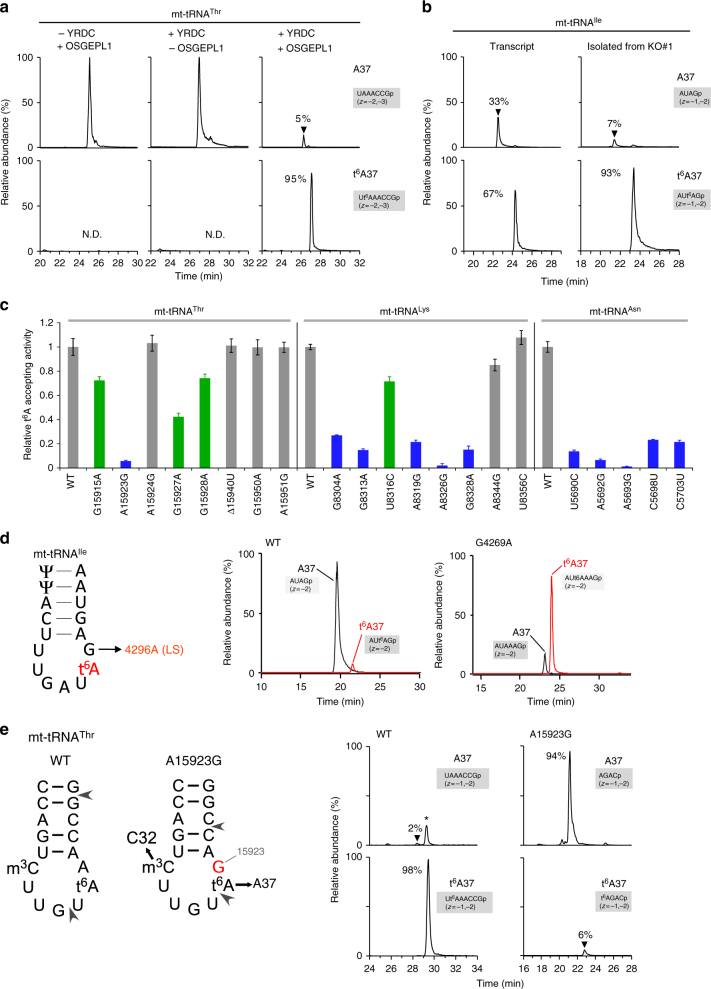
In vitro reconstitution of t^6^A37 on mt-tRNAs and impairment of t^6^A37 by pathogenic point mutations associated with mitochondrial diseases. **a** In vitro formation of t^6^A37 on mt-tRNA^Thr^ transcripts with or without YRDC and OSGEPL1 in the presence of Thr, ATP, and bicarbonate. After the reaction, mt-tRNA^Thr^ was digested with RNase T_1_ and analyzed by LC/MS. XICs generated by integration of multiply-charged negative ions of A37-containing fragments of mt-tRNA^Thr^ harboring A37 (top) and t^6^A37 (bottom) (Supplementary Table 1). Intensity fractions (%) of modified or unmodified fragments are denoted. N.D., not detected. **b** In vitro formation of t^6^A37 on mt-tRNA^Ile^ transcript or native mt tRNA^Ile^ isolated from *OSGEPL1* KO#1 cells. XICs generated by integration of multiply-charged negative ions of A37-containing fragments of mt-tRNA^Ile^ harboring A37 (top) and t^6^A37 (bottom) (Supplementary Table 1). Intensity fractions (%) of modified or unmodified fragments are denoted. **c** Efficiency of t^6^A37 formation in mt-tRNA mutants. The numbering of each mutant corresponds to that in Fig. 1b. Bars corresponding to severe and mild reduction in t^6^A37 formation are colored in blue and green, respectively. **d** G4296A in mt-tRNA^Ile^ (left panel) is a unique pathogenic mutation that promotes t^6^A37 formation. XICs show A37-containing fragments of WT and G4296A mutant mt-tRNA^Ile^ harboring A37 (black) and t^6^A37 (red). *m/z* values of each fragment are listed in Supplementary Table 1. **e** Impairment of t^6^A37 in mt-tRNA^Thr^ with A15923G mutation isolated from the myoblasts of a patient with MERRF-like symptoms. Anticodon stem-loop sequences of WT (left) and A15923G mutant (right) mt-tRNA^Thr^. Positions of RNase T_1_ and RNase A digestion are indicated by arrowheads. m^3^C32 in the A15923G mutant is partially converted to C32 (Supplementary Fig. 5b). XICs generated by integration of multiply-charged negative ions of A37-containing fragments of mt-tRNA^Thr^ harboring A37 (upper panels) and t^6^A37 (lower panels) (Supplementary Table 1) from WT (left panels) and A15923G myoblasts (right panels). Intensity fractions (%) of modified or unmodified fragments are indicated

To determine how OSGEPL1 recognizes these mt-tRNAs with diverse structures, we established an in vitro reconstitution system for mitochondrial t^6^A formation and examined its substrate specificity. Soluble YRDC and OSGEPL1 were recombinantly expressed in *E. coli*. As tRNA substrates, we prepared five species of human mt-tRNAs for Thr, Asn, Ile, Ser(AGY), and Lys by in vitro transcription. These tRNAs were incubated with recombinant YRDC (17–279a.a.) and OSGEPL1 (35–414a.a.) in the presence of Thr, ATP, and bicarbonate. The products were digested by RNase T_1_ and subjected to RNA-MS. We detected t^6^A-containing fragments only in the presence of both enzymes (Fig. 5a, b and Supplementary Fig. 8a, b). t^6^A37 was efficiently introduced into mt-tRNAs for Thr (96%) (Fig. 5a), Asn (98%), and Lys (95%) (Supplementary Fig. 8a). By contrast, efficiency of t^6^A37 formation was lower in the mt-tRNAs for Ile (67%) (Fig. 5b) and Ser(AGY) (34%) (Supplementary Fig. 8b). We considered that this outcome might be due to the lack of other modifications in tRNA transcripts. Accordingly, we isolated individual mt-tRNA^Ile^ and mt-tRNA^Ser(AGY)^ lacking t^6^A37 from *OSGEPL1*-KO cells and examined t^6^A37 formation in these tRNAs in vitro. The frequency of t^6^A37 formation was markedly increased (from 67 to 93%) in mt-tRNA^Ile^ (Fig. 5b), but slightly increased (from 34 to 48%) in mt-tRNA^Ser(AGY)^ (Supplementary Fig. 8b). This result suggests that modifications in other regions contribute to the efficiency of t^6^A37 formation in these mt-tRNAs.

To characterize mitochondrial t^6^A37 formation, we examined the activity of *E. coli* t^6^A-modifying enzymes (YrdC, YgjD, YjeE, and YeaZ) toward the five mt-tRNAs under the same assay conditions. The *E. coli* system introduced t^6^A37 efficiently in mt-tRNAs for Thr (93%), Asn (94%), and Lys (90%) (Supplementary Fig. 8c), but much less efficiently in mt-tRNAs for Ile (6%) and Ser(AGY) (7%) (Supplementary Fig. 8c), indicating that the *E. coli* system has substrate specificity for t^6^A37 formation. Together, these findings indicate that human OSGEPL1 evolved to acquire broad substrate specificity to accommodate at least five mt-tRNAs with diverse structures.

We found several isoforms of YRDC with different N-termini. To compare their activities, we prepared three YRDC isoforms (full length, 17–279a.a. and 52–279a.a.) (Fig. 2c), and compared their t^6^A-forming activity using mt-tRNA^Asn^ as a substrate. They all showed efficient activity with little significant difference between three isoforms (Supplementary Fig. 9).

### Pathogenic mutations impair mitochondrial t^6^A37 formation

Pathogenic mutations in mtDNA are associated with mitochondrial disorders (MITOMAP: http://mitomap.org/MITOMAP). Among them, about 200 mutations reside in tRNA genes. Curiously, pathogenic mutations in each tRNA are linked to different symptoms of mitochondrial diseases. Given that t^6^A37 plays critical roles in mitochondrial protein synthesis, we speculated that some of the pathogenic point mutations in mt-tRNA genes impair t^6^A37 formation, in turn leading to mitochondrial dysfunction. From among the previously described pathogenic mutations, we prepared 21 mutant mt-tRNAs for Thr, Lys, and Asn (Fig. 1b) by in vitro transcription and then examined their ability to undergo t^6^A37 modification mediated by YRDC and OSGEPL1 (Fig. 5c). As expected, two of the target-site mutations, A8326G in mt-tRNA^Lys^ and A5693G in mt-tRNA^Asn^, completely abolished t^6^A37 formation. Among eight mutants of mt-tRNA^Thr^, only A15923G strongly inhibited t^6^A37 formation, whereas three mutations, G15915A, G15927A, and G15928A, had a milder effect with relative activity 0.4–0.8. In mt-tRNA^Lys^, four mutations (G8304A, G8313A, A8319G, and G8328A) caused severe reductions in t^6^A37 formation with relative activity less than 0.3. In mt-tRNA^Asn^, t^6^A37 formation was markedly impaired by four mutations (C5703U, C5698U, A5692G, and U5690C) with relative activity less than 0.3. Collectively, among 21 pathogenic point mutations in three mt-tRNA genes, two target-site mutations and nine mutations strongly affected t^6^A37 formation, indicating that loss of t^6^A37 is a major cause of mitochondrial disorders. Multiple mutations that reduced t^6^A37 reside in the anticodon arm of mt-tRNAs (Fig. 1b), suggesting that OSGEPL1 primarily recognizes the anticodon step-loop structure to introduce t^6^A37.

Because we detected little t^6^A37 formation in mt-tRNA^Thr^ bearing the A15923G mutation, as well as in mt-tRNA^Asn^ bearing the A5692G mutation (Fig. 5c), we concluded that A38 is a strong determinant for t^6^A37 formation and that A-to-G mutation in this position strongly impaired t^6^A37 formation. Nevertheless, human mt-tRNA^Ile^ contains G38 (Fig. 1b), explaining why mt-tRNA^Ile^ is a poor substrate for OSGEPL1 as observed in Fig. 5b. Notably in this regard, a G-to-A pathogenic mutation at position 38 (G4296A) is associated with Leigh syndrome (Figs. 1b and 5d)^48^. We prepared in vitro transcripts of mt-tRNA^Ile^ bearing the G4296A mutation and examined its ability to undergo t^6^A37 formation. The G4296A mutation significantly promoted t^6^A37 formation (Fig. 5d), suggesting that the G38 residue in WT mt-tRNA^Ile^ plays a regulatory role in preventing t^6^A37 formation.

### Hypomodification of t^6^A37 in mt-tRNA in patient cells

To verify the loss of t^6^A37 in mutant mt-tRNAs in patient cells, we obtained fibroblasts and myoblasts harboring the A15923G mutation from a 12-year-old female patient with symptoms of MERRF, lactic acidosis, short stature, and hearing loss. Because clinical specimens are heteroplasmic with respect to mtDNA, we first determined the mtDNA mutation rate using the restriction fragment length polymorphism (RFLP method (Supplementary Fig. 10a)). The A15923G mutation rate in fibroblasts and myoblasts was 32% and 62%, respectively (Supplementary Fig. 10b). We then isolated mt-tRNA^Thr^, along with mt-tRNA^Ser(AGY)^ and ct-tRNA^Ile(IAU)^, from the mutant cells. Because the isolated mt-tRNA^Thr^ contained a mixture of WT and A15923G mutant tRNAs, we analyzed these two tRNAs separately by digesting with two different RNases. For WT mt-tRNA^Thr^, we detected a t^6^A37-containing heptamer fragment (positions 36–42) generated by RNase T_1_ digestion (Fig. 5e). For mt-tRNA^Thr^ harboring the A15923G mutation we used RNase A to detect tetramer fragments (positions 37–40) to measure t^6^A37 frequency (Fig. 5e). We observed low levels of t^6^A37 in myoblasts (6%) (Fig. 5e) and fibroblasts (5%) (Supplementary Fig. 10c), whereas WT mt-tRNA^Thr^ was completely modified by t^6^A37 in both cell types (Fig. 5e and Supplementary Fig. 10c). As a control, we confirmed no reduction in t^6^A37 in mt-tRNA^Ser(AGY)^ and ct-tRNA^Ile^ isolated from patient fibroblasts (Supplementary Fig. 10d) and myoblasts (Supplementary Fig. 10e).

Taken together, these results are consistent with our in vitro mutation studies and demonstrate that pathogenic point mutations in mtDNA impaired t^6^A37 formation of mt-tRNA in patient cells, strongly indicating that the lack of t^6^A37 contributes to molecular pathogenesis of mitochondrial disease.

Because t^6^A37 is required for efficient m^3^C32 formation (Supplementary Fig. 5a), we measured the frequency of m^3^C32 in mt-tRNA^Thr^ isolated from the patient’s cells; the results revealed 44% in fibroblasts and 40% in myoblasts (Supplementary Fig. 5b). Thus, hypomodification at position 32 caused by loss of t^6^A37 might be an additional factor that contributes to mitochondrial disorders.

### Mitochondrial t^6^A37 is sensitive to intracellular CO_2_

**Fig. 6:**
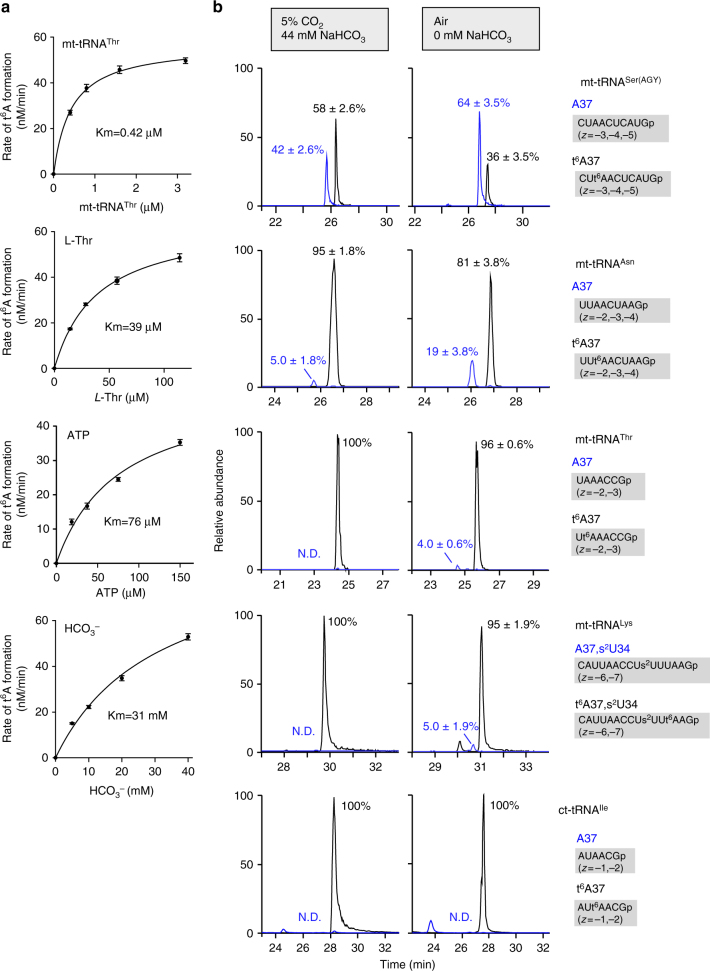
Mitochondrial t^6^A37 formation is sensitive to intracellular bicarbonate concentration. **a** Kinetic analyses of mitochondrial t^6^A37 formation mediated by YRDC and OSGEPL1. Initial velocities of t^6^A37 formation were measured against variable concentrations of mt-tRNA^Thr^, *L*-Thr, ATP, and bicarbonate. Km values for each substrate are indicated. **b** Hypomodification of t^6^A37 in mt-tRNAs in HEK293T cells cultured in non-bicarbonate medium. XICs generated by integration of multiply-charged negative ions of A37-containing fragments of mt-tRNA^Ser(AGY)^ (top panels), mt-tRNA^Asn^ (second panels), mt-tRNA^Thr^ (third panels), mt-tRNA^Lys^ with s^2^U34 (fourth panels) and ct-tRNA^Ile^ (bottom panels) bearing A37 (blue) and t^6^A37 (black) (Supplementary Table 1) isolated from HEK293T cells cultured with normal DMEM medium (44 mM NaHCO_3_) in 5% CO_2_ (left panels) and non-bicarbonate medium in air (right panels). mt-tRNA^Ser(AGY)^ and other tRNAs were isolated from the cells cultured for 6 and 3 days, respectively. t^6^A frequencies (%) are described as mean values ± s.d. of technical triplicate

We next measured the kinetic parameters for mitochondrial t^6^A37 formation on mt-tRNA^Thr^, and determined apparent Km values for each substrate (Fig. 6a and Supplementary Table 2). The Km values for mt-tRNA^Thr^ were sub-micromolar (0.42 µM), indicating that OSGEPL1 has sufficient affinity for the tRNA substrate during t^6^A37 formation. By contrast, the Km values for ATP and *L*-Thr were 76 μM and 39 μM, respectively. Strikingly, the Km value (31 mM) for bicarbonate was quite high, but within the range of bicarbonate concentration (10–40 mM) in human mitochondria^49^, indicating that mitochondrial t^6^A37 formation is sensitive to cellular bicarbonate concentration. Because bicarbonate is a substrate for TC-AMP formation catalyzed by YRDC^32^, we also measured the kinetic parameters for this reaction (Supplementary Table 3). The Km value for this reaction was 13 mM, demonstrating that mitochondrial TC-AMP formation also requires a high cellular concentration of bicarbonate.

For cell culture, we generally use DMEM medium, which contains 44 mM sodium bicarbonate, in a 5% CO_2_ atmosphere, which should provide a sufficient concentration of bicarbonate in mitochondria. Indeed, under these conditions, high t^6^A frequency was observed in mt-tRNAs isolated from HEK293T cells (Figs. 2e and 3e, Supplementary Fig. 4). To determine whether the t^6^A37 frequency is regulated by bicarbonate concentration, we cultured HEK293T cells in medium without sodium bicarbonate in the absence of 5% CO_2_ (air atmosphere) (Supplementary Fig. 11). Subsequently, four species of mt-tRNAs, along with ct-tRNA^Ile^, were isolated and subjected to RNA-MS (Fig. 6b). The t^6^A37 frequency of mt-tRNA^Ser(AGY)^ decreased markedly from 58 ± 2.6% to 36 ± 3.5% (*P* = 0.00018) upon CO_2_-depletion. We also found significant decrease of t^6^A frequency in mt-tRNA^Asn^ from 95 ± 1.8% to 81 ± 3.8% (*P* = 0.00432). Slight reduction of t^6^A was observed in mt-tRNA^Lys^ and mt-tRNA^Thr^ (Fig. 6b and Supplementary Fig. 12). However, no reduction of t^6^A was observed in ct-tRNA^Ile^ under CO_2_-depleted conditions. The different responses of t^6^A37 formation in these mt-tRNAs to bicarbonate concentration might be due to differences in efficiency of t^6^A37 formation. As shown previously (Supplementary Fig. 8b), mt-tRNA^Ser(AGY)^ is a poorer substrate for OSGEPL1 than the other mt-tRNAs. Consistent with this, the rate of t^6^A37 formation of mt-tRNA^Ser(AGY)^ was much slower than that of mt-tRNA^Thr^ (Supplementary Fig. 13a). For mt-tRNA^Ser(AGY)^, we monitored the t^6^A37 formation at increasing concentrations of bicarbonate (20–40 mM), revealing that the rate of t^6^A37 formation increased in response to the bicarbonate concentration (Supplementary Fig. 13b). Inefficient t^6^A37 formation in this mt-tRNA could be supported by higher concentrations of TC-AMP. This speculation provides a kinetic explanation for the observation that mt-tRNA^Ser(AGY)^ is sensitive to cellular bicarbonate concentration.

Rapidly growing tumors frequently fall into hypoxia due to low oxygen availability^50^. Given that mitochondrial CO_2_/bicarbonate is largely provided by TCA cycle, we asked whether mitochondrial t^6^A37 could be affected by hypoxic conditions in solid tumors. For these experiments, we prepared solid tumor xenografts by subdermally injecting colorectal adenocarcinoma HT-29 cells into nude mice, and then isolated and mt-tRNA^Ser(AGY)^ along with ct-tRNA^Ile^ for RNA-MS analyses. As shown in Supplementary Fig. 14, we detected hypomodification of t^6^A37 in mt-tRNA^Ser(AGY)^ isolated from the tumor, whereas high t^6^A frequency was observed in the same tRNA isolated from cell culture. This finding implies that t^6^A37 frequency can be modulated by hypoxic conditions in solid tumors.

## Discussion

Here, we showed that YRDC and OSGEPL1 are required for t^6^A37 formation in mt-tRNAs. YRDC is predominantly localized in the cytoplasm, where it provides TC-AMP to the KEOPS complex to synthesize t^6^A37 in cytoplasmic tRNAs. However, YRDC contains a weak N-terminal MTS, and a certain fraction of YRDC is transported to mitochondria. In budding yeast, the YRDC homolog Sua5p is also localized in both cytoplasm and mitochondria^51^. A longer isoform of Sua5p translated from the upstream AUG codon has an MTS and enters mitochondria, while the smaller isoform translated from the downstream AUG codon at position 10 is predominantly localized in the cytoplasm. Although human YRDC also has the downstream AUG codon, it is not conserved among mammals (Supplementary Fig. 1a), indicating that the dual localization of YRCD in mammals and yeast operate via different mechanisms.

In *OSGEPL1* KO strains, no t^6^A37 was formed in any of the five species of mt-tRNAs examined (Fig. 3e and Supplementary Fig. 4), although the steady-state levels of these mt-tRNAs were unchanged (Supplementary Fig. 7). The KO cells did not grow well in galactose medium (Fig. 4a), indicating minimal mitochondrial activity in these strains. Pulse-labeling experiments revealed that mitochondrial protein synthesis was markedly decreased by the absence of t^6^A37 (Fig. 4f). In particular, we observed severe reduction of protein synthesis for Complex I components, including ND1, ND2, ND4, ND5, and ND6 (Fig. 4f). In fact, steady-state levels of ND2 and ND5 decreased in KO cells (Fig. 4e). Analysis of codon usage in 13 proteins encoded by mtDNA (Supplementary Table 4) revealed that the frequencies of the codons read by the five mt-tRNAs bearing t^6^A37 were higher in ND2 and ND5 than in the other genes. Thus, the lack of t^6^A37 reduces the decoding ability of the five mt-tRNAs, causing codon-specific dysfunction in mitochondrial translation. In addition, the lysylation level of mt-tRNA^Lys^ was reduced in KO cells (Fig. 4g), indicating that t^6^A37 is required for efficient aminoacylation by lysyl-tRNA synthetase (KARS). Although the pleiotropic functions of t^6^A have been extensively studied in bacteria and yeast, this study provides the first evidence for a physiological role of t^6^A37 in human cells.

We successfully reconstituted t^6^A37 formation in vitro using recombinant YRDC and OSGEPL1. OSGEPL1 efficiently employed all five species of mt-tRNAs as substrates (Fig. 5a, b and Supplementary Fig. 8a, b), whereas *E. coli* enzymes only recognized three of the mt-tRNAs (Supplementary Fig. 8c). This observation indicates that OSGEPL1 has a broad substrate specificity that can accommodate the five mt-tRNAs with non-canonical clover leaf structures. However, in comparison with the other three substrates, mt-tRNA^Ser(AGY)^ and mt-tRNA^Ile^ were poor substrates for t^6^A37 formation (Fig. 5b and Supplementary Fig. 8b), indicating that OSGEPL1 is less able to recognize these two tRNAs. Because mt-tRNA^Ile^ lacking t^6^A37 isolated from *OSGEPL1* KO cells was a better substrate for t^6^A37 formation than the in vitro transcribed mt-tRNA^Ile^ (Fig. 5b), it is likely that one or a combination of other modifications, m^1^G9, m^2,2^G26, Ψ27, and Ψ28, in mt-tRNA^Ile^ (Fig. 1b) supports efficient t^6^A37 formation mediated by OSGEPL1. The observation that each mt-tRNA exhibited a different t^6^A37 formation activity implies that mitochondrial translation is regulated in a codon-specific manner.

Pathogenic mutations in mt-tRNAs associated with mitochondrial diseases frequently impair post-transcriptional modifications in mt-tRNAs, resulting in dysfunction of mitochondrial protein synthesis. We previously reported the absence of τm^5^U34 in mt-tRNA^Leu(UUR)^ with each of five pathogenic point mutations associated with MELAS, and the absence of τm^5^s^2^U34 in mt-tRNA^Lys^ with one pathogenic mutation associated with MERRF^7^. In addition, we found two pathogenic point mutations that impair f^5^C34 formation in mt-tRNA^Met52^^52^. In this study, we examined the impact of 22 pathogenic point mutations in three mt-tRNAs on t^6^A37 formation in vitro and found 15 mutations that impaired or reduced t^6^A37 formation (Fig. 5c). As expected, we detected little t^6^A37 formation in mt-tRNA^Lys^ bearing the A8326G mutation or in mt-tRNA^Asn^ bearing the A5693G mutation because these two variants are target site mutations at position 37 (Fig. 1b). Similarly, no t^6^A37 would be expected in mt-tRNA^Ile^ bearing the target site mutation (A4295G). Many mutations that impaired t^6^A37 formation reside in the anticodon stem-loop of mt-tRNAs. In mt-tRNA^Lys^, mutations in the D-stem also affected t^6^A37 formation, indicating that OSGEPL1 recognizes the sequence and/or structure of the anticodon stem-loop and D-stem in mt-tRNAs. In particular, A15923G in mt-tRNA^Thr^ and A5692G in mt-tRNA^Asn^ strongly impaired t^6^A37 formation. Because both mutations are located at position 38, A38 (which is 3′ adjacent to the target site) (Fig. 1b) acts as a positive determinant for t^6^A37 formation. Indeed, we found little t^6^A37 in mt-tRNA^Thr^ with the A15923G mutation isolated from the patient’s cells with MERRF-like symptoms (Fig. 5e and Supplementary Fig. 10c). These results demonstrate that pathogenic point mutations in mt-tRNAs inhibit t^6^A37 formation, leading to deficient decoding activity and tRNA function in mitochondrial protein synthesis.

Intriguingly, mt-tRNA^Ile^ naturally contains G38 (G4296) (Fig. 1b) which should work as a negative determinant for t^6^A37 formation, as observed in A-to-G pathogenic point mutations at the corresponding positions in mt-tRNA^Thr^ and mt-tRNA^Asn^. This feature nicely explains why mt-tRNA^Ile^ is a poor substrate for OSGEPL1. G4296A has been reported as a pathogenic mutation associated with Leigh syndrome, juvenile Parkinsonism, and hypogonadism^48, 53, 54^. Here, we revealed that this pathogenic mutation markedly improved t^6^A37 formation of mt-tRNA^Ile^ in vitro (Fig. 5d), suggesting that G38 has an attenuator function that regulates t^6^A37 frequency. Under normal culture conditions, mt-tRNA^Ile^ has a higher frequency (over 90%) of t^6^A37 (Fig. 2e and Supplementary Fig. 4), indicating that other modifications might contribute to efficient recognition by OSGEPL1 (Fig. 5b). Among 282 mtDNAs in mammalian species reported so far, A37 and G38 in mt-tRNA^Ile^ are conserved in 280 species^55^, suggesting that the regulatory effect of G38 on t^6^A37 formation plays a physiological role in certain biological contexts and conditions.

**Fig. 7:**
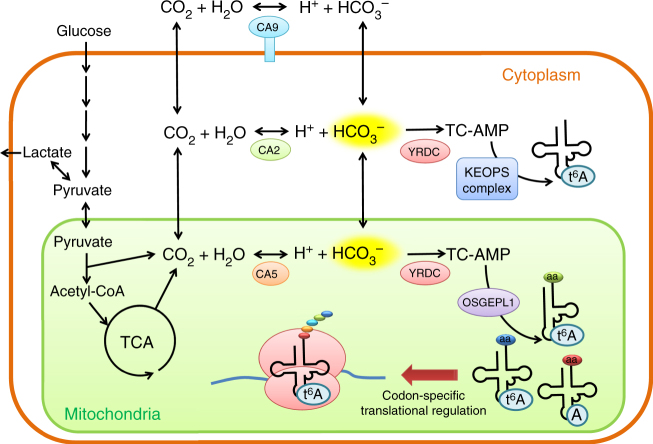
Metabolism of bicarbonate and t^6^A37 formation in cytoplasm and mitochondria. CO_2_ produced by TCA cycle is hydrated to form bicarbonate by carbonic anhydrase 5 (CA5) in mitochondria. In addition, mitochondrial CO_2_ is exported to cytoplasm and hydrated by carbonic anhydrase 2 (CA2). In hypoxic conditions, carbonic anhydrase 9 (CA9) is overexpressed on the cell surface by HIF-1 pathway. CA9 generates large amounts of bicarbonate outside the cell. Then, cells actively import extracellular bicarbonate to neutralize lactate and prevent acidification. YRDC employs bicarbonate to synthesize TC-AMP, which is used for t^6^A37 formation on tRNAs mediated by KEOPS complex in cytoplasm and by OSGEPL1 in mitochondria. Hypomodification of t^6^A37 in two mt-tRNAs in the cells cultured without bicarbonate, as well as from hypoxic solid tumors, indicating codon-specific translational regulation by sensing intracellular bicarbonate

Kinetic studies of t^6^A37 formation revealed that the Km value of bicarbonate (31 mM) was extremely high (Fig. 6a and Supplementary Table 2), whereas the Km values of other substrates required for t^6^A37 formation were in the micromolar range. For TC-AMP formation catalyzed by YrdC, *L*-Thr needs to form a carbamate (*N*-carboxy-*L*-Thr), followed by adenylation^32^. It is known that amino acids form carbamates at high concentration of bicarbonate or CO_2_ at alkaline pH non-enzymatically^56^. The high Km value of bicarbonate for TC-AMP and t^6^A37 formation can be explained by the carbamate formation of *L*-Thr at the initial step of the reaction. Considering that mitochondrial bicarbonate concentration ranges from 10 to 40 mM^49^ ([S]/Km for bicarbonate = 0.32–1.3), the frequency of t^6^A37 in mt-tRNAs should be regulated by intracellular bicarbonate concentrations. When HEK293T cells were cultured with non-bicarbonate medium in an air atmosphere, the levels of t^6^A37 in mt-tRNAs were reduced (Fig. 6b). Because ct-tRNA^Ile^ retained a high frequency of t^6^A37 under this culture condition, bicarbonate-sensitive t^6^A37 formation is specific for mt-tRNAs. Although most of the mitochondrial bicarbonate is assumed to originate from CO_2_ produced by respiration in the mitochondria, extracellular bicarbonate supplied in the medium is required to maintain high t^6^A37 frequency in mt-tRNAs. HeLa cells require a sufficient concentration of bicarbonate in the medium to engage in rapid proliferation^57^, indicating that respiratory CO_2_ in mitochondria is not sufficient to support cell growth. Considering that t^6^A37 has pleiotropic functions in protein synthesis, we presume that bicarbonate-sensitive t^6^A37 formation contributes to codon-specific translational regulation in mitochondria via sensing of respiratory activity and extracellular bicarbonate concentration. In particular, when mitochondrial respiration decreases, intake of extracellular CO_2_ or bicarbonate is necessary to support cell viability (Fig. 7). Rapidly proliferating solid tumors frequently outgrow their blood supply, leading to a hypoxic microenvironment in the inner region of the tumor due to low oxygen availability^58^. In this situation, the mitochondrial concentration of bicarbonate should be reduced because TCA cycle is inhibited^59^. In fact, we observed hypomodified t^6^A37 in mt-tRNA^Ser(AGY)^ isolated from HT-29 xenografts (Supplementary Fig. 14), implying that mitochondrial bicarbonate is not sufficient to support efficient t^6^A37 formation in mt-tRNAs in hypoxic tumors. Under hypoxic conditions, HIF-1α is stabilized and activates a set of genes related to anaerobic glycolysis^60^, resulting in massive accumulation of lactate in the cell. In addition, carbonic anhydrase 9 (CA9) is overexpressed on the cell surface as a consequence of HIF-1α stabilization, and plays a critical role in generating large amounts of bicarbonate and protons (Fig. 7)^61^. Cells actively take up extracellular bicarbonate to neutralize lactate and prevent acidification; in addition, bicarbonate is used to support high frequency of t^6^A37 in cytoplasmic tRNAs (Fig. 6b and Supplementary Fig. 14). At the same time, mitochondria are thought to import bicarbonate from cytoplasm to maintain levels of t^6^A37 in mt-tRNAs (Fig. 7). However, we clearly detected hypomodification of t^6^A37 in mt-tRNAs (Fig. 6b and Supplementary Fig. 14), indicating that mitochondrial t^6^A37 formation serves as a sensor function to monitor hypocarnia conditions. Given that hypomodification of t^6^A37 in mt-tRNAs downregulates mitochondrial translation at codon-specific manner, CO_2_ sensitivity of t^6^A37 might be a part of Warburg effect^62^ to control oxidative phosphorylation (OXPHOS) activity via translational regulation in mitochondria under hypoxic condition.

In this study, we showed that YRDC and OSGEPL1 are required for t^6^A37 formation in five mt-tRNAs and that t^6^A37 plays critical roles in protein synthesis and respiratory activities in mitochondria. Kinetic studies revealed that mitochondrial t^6^A37 formation is dynamically regulated by cellular bicarbonate concentration. In support of this finding, we observed hypomodification of t^6^A37 in mt-tRNA isolated from human cells cultured with non-bicarbonate medium, as well as hypoxic solid tumors, implying codon-specific regulatory translation under hypoxic conditions. We also identified pathogenic point mutations that impaired t^6^A37 formation in mt-tRNAs and confirmed low levels of t^6^A37 in mt-tRNA^Thr^ with the A15923G mutation isolated from a patient with MERRF-like symptoms, indicating that the lack of t^6^A37 has pathological consequences.

## Methods

### Cell culture and growth measurement

HEK293T, HeLa, HT-29, fibroblast, and myoblast cells were cultured at 37 °C in 5% CO_2_ in DMEM (D5796, Sigma) supplemented with 10% or 20% FBS (Gibco). The viable cell numbers were counted by trypan blue staining assay.

Growth of HEK293T cells was measured using AlamarBlue (Invitrogen) in glucose medium (Wako DMEM 042–32255 supplemented with 10% FBS and 1 mg/mL glucose) or galactose medium (Wako DMEM 042–32255 supplemented with 10% FBS and 1 mg/mL galactose). Cells were inoculated at a density of 2.0 × 10^3^ cells/well on collagen I-coated 96-well plates (Iwaki). On each day of cell cultivation (days 0 to 6), 1/10 volume of AlamarBlue reagent was added to each well and incubated for 4 h; absorbance was then measured at 570 nm and 600 nm using a SpectraMax 190 microplate reader (Molecular Devices).

For growth measurement in non-bicarbonate medium, bicarbonate-free DMEM (D5648, Sigma) was supplemented with 10% FBS (Gibco), 30 mM HEPES (Rikaken), and 10 μg/mL each of nucleosides (adenosine, cytidine, guanosine, and uridine), and adjusted to pH 7.1 with NaOH. To add back bicarbonate, the non-bicarbonate medium was supplemented with 44 mM NaHCO_3_, and adjusted to pH 7.2 with HCl. HEK293T cells precultured in DMEM were suspended in the non-bicarbonate medium. Cells were seeded at a density of 4 × 10^3^ cells/well on 96-well cell culture plates (TrueLine), and cultured at 37 °C under air (~0.04% CO_2_) for the non-bicarbonate medium, and under 5% CO_2_ for the bicarbonate-added back medium, respectively. On each day of cell culture, 1/10 volume of 1.4 mM resazurin sodium salt (Nacalai Tesque) dissolved in PBS was added to each well and incubated for 3 h. Fluorescence was then measured with excitation/emission at 550/600 nm using an Infinite 200 Pro M Plex (TECAN).

To estimate CO_2_ effect on t^6^A frequency, HEK293T cells were cultured in the non-bicarbonate medium with or without 44 mM NaHCO_3_ as described above, under 5% CO_2_ or air (~0.04% CO_2_), respectively, at 37 °C for 3 days. For mt-tRNA^Ser(AGY)^, prolonged cultivation was conducted as following. The cells were seeded in 15 cm dishes and pre-cultured to 70% confluence at 37 °C in 5% CO_2_ in DMEM (D5796, Sigma) supplemented with 10% FBS (Gibco). Then, the cells were washed twice with non-bicarbonate medium consisted of bicarbonate-free DMEM (D5648, Sigma), 10% FBS (Gibco), 30 mM HEPES-NaOH (pH 7.4), and 10 μg/mL each of nucleotides (adenosine, cytidine, guanosine, and uridine) (final, pH 7.2), and then cultured with the same medium at 37 °C without CO_2_ (air atmosphere) for 6 days. The medium was exchanged every 3 days. Total RNA was extracted, individual tRNAs were isolated, and tRNA modifications were analyzed by MS as described below.

### Construction of knockout cell lines

*OSGEPL1* KO cells were generated using the CRISPR/Cas9 system as described previously^52, 63^. Sense and antisense oligonucleotides for a guide RNA (sgRNA) (Supplementary Data 1) were cloned into vector pX330 (Addgene plasmid 42230)^64^. HEK293T cells were transfected with a pX330 vector bearing the sgRNA sequence and with pEGFP-N1 (Clontech) and pLL3.7 vectors containing the puromycin resistance gene; transfections were performed using FuGENE HD (Promega). One day after transfection, cells were seeded at low density, and transfectants selected with 1 μg/mL puromycin. Knockout lines were selected by the surveyor assay. The target region of the genome in each clone was PCR-amplified using primers listed in Supplementary Data 1 and sequenced.

### RNA extraction and isolation of individual tRNAs

Total RNA from culture cells was extracted using TriPure Isolation Reagent (Roche). For acid-urea PAGE-northern blotting^65^ to detect aminoacylation levels of tRNAs, total RNA was obtained by phenol extraction under acidic conditions using 50 mM NaOAc (pH 5.3) at 4 °C.

Individual tRNAs were isolated by reciprocal circulating chromatography as described^22, 44^ using the DNA probes listed in Supplementary Data 1.

### Mass spectrometry

Liquid chromatography-mass spectrometry (LC/MS) analyses of tRNA fragments were performed as described previously^22, 45^. Around 1 pmol of each isolated tRNA was digested at 37°C for 30 min with 50 units RNase T_1_ (Thermo Scientific) in 20 mM NH_4_OAc (pH 5.3) or 10 ng RNase A (Ambion) in 20 mM NH_4_OAc (pH 7.7), followed by addition of an equal volume of 0.1 M triethylamine acetate (TEAA; pH 7.0). The digest (800 fmol) was subjected to the trap column for desalting and chromatographed by a HiQ sil C18W-3 nano spray column (C18, 3 μm, 120 Å pore size; ID 0.1 × 100 mm, KYA Technologies) with solvent system consisted of 0.4 M 1,1,1,3,3,3-hexafluoro-2-propanol (HFIP) (pH 7.0) (solvent A) and 0.4 M HFIP (pH 7.0) in 50% methanol (solvent B) at a flow rate of 300 nL/min with a linear gradient of 5–100% B over 35 min with a splitless nano HPLC system (DiNa, KYA Technologies). The eluent was ionized by ESI source, and directly introduced into an iontrap-orbitrap hybrid mass spectrometer (LTQ Orbitrap XL, Thermo Fisher Scientific). Ions were scanned with a negative polarity mode over an *m/z* range of 600–2000 throughout the separation. Exact molecular mass and its multiply-charged negative ions of the t^6^A37-containing RNA fragment in each tRNA was calculated by accurate atomic composition. Modification frequency was calculated by peak area ratio of mass chromatograms of modified versus unmodified fragments.

For estimation of ESI detection efficiencies of RNA fragments with and without t^6^A modification, human mt-tRNA^Ile^ isolated from WT HEK293T cells and its unmodified one isolated from *OSGEPL1* KO cells (KO#2) were mixed with different ratio (total quantity is adjusted to 1 pmol in each sample), digested by RNase T_1_ and subjected to LC/MS. The peak area ratio of the A37-containing fragments with and without t^6^A was plotted against the mixing ratio for each sample (Supplementary Fig. 3). Then, t^6^A frequency of the native mt-tRNA^Ile^ was calculated with the slope of this plot, and the mixing ratios were transformed to t^6^A frequencies. The resulting plot of t^6^A frequency versus intensity ratio is depicted in Supplementary Fig. 3.

YRDC or OSGEPL1, immunoprecipitated with an anti-FLAG-tag antibody, was resolved by SDS-PAGE. Cys residues were alkylated by iodoacetic acid, followed by digestion with sequencing grade trypsin or AspN (Promega) using an in-gel digestion method. The digested peptides were then subjected to capillary LC/nano ESI-MS as previously described^66^.

### Expression and purification of recombinant proteins

For protein expression in HeLa or HEK293T cells, cDNAs of *YRDC* (NM_024640.3) and *OSGEPL1* (NM_022353.2) were amplified by RT-PCR with sets of primers (Supplementary Data 1) and cloned into vector pDEST12.2 with a C-terminal FLAG-tag using the pENTR/D-topo Gateway cloning kit (Invitrogen). Transfection of plasmids was conducted using FuGENE HD reagent (Promega) or linear polyethylenimine.

For construction of recombinant proteins, three isoforms of YRDC (full length, 17–279a.a. and 52–279a.a.) were cloned into vector pE-SUMO using the indicated primers (Supplementary Data 1). The recombinant YRDC fused with N-terminal His6-SUMO was overexpressed in the *E. coli* Rosetta (DE3) strain (Novagen) cultured in LB medium and induced with 10 μM IPTG at 22 °C overnight. The N-terminal His6-SUMO tagged YRDC was purified from the cell lysate using a HisTrap HP column (GE Healthcare) on an AKTA purifier 10 system (GE Healthcare). Recombinant protein was digested with TEV protease (Promega) and applied on the HisTrap column to remove the His_6_-SUMO tag. The flow-through fraction was dialyzed in buffer containing 50 mM HEPES-KOH (pH 7.5), 100 mM KCl, 2 mM MgCl_2_, and 1 mM DTT, followed by addition of glycerol (final concentration, 20%) and storage at −30 °C.

OSGEPL1 (35–414a.a.) was cloned into pET28a (Novagen) using a set of primers (Supplementary Data 1). The C-terminal His6-tagged OSGEPL1 was overexpressed in the *E. coli* Rosetta (DE3) strain (Novagen) cultured in LB medium and induced with 100 μM IPTG at 28 °C overnight. Soluble OSGEPL1 was trapped on a HisTrap HP column (GE Healthcare), further purified on a MonoQ column (GE healthcare), and stored as described above.

### Western blotting and antibodies

Mitochondrial fractions were isolated from WT and *OSGEPL1* KO HEK293T cells (1 × 10^7^ cells) using the Mitochondria Isolation Kit (Miltenyi Biotec. K.K.) and subjected to western blotting to measure steady-state levels of protein components in respiratory chain complexes using the Total OXPHOS Rodent WB Antibody Cocktail (an antibody mixture targeting ATP5A, UQCRC2, MTCO1, SDHB, and NDUF88; ab110413, Abcam), an anti-ND5 antibody (ab92624, Abcam), and an anti-ND2 antibody (19704-1-AP, Proteintech). Other antibodies used in this study were as follows: anti-OSGEPL1 (25694-1-AP, Proteintech), anti-GAPDH (6C5, Santa Cruz Biotech), anti-FLAG-tag (1E6, Wako), HRP-conjugated donkey anti-mouse/rabbit IgG (715-035-150/715-035-152, Jackson ImmunoResearch), anti-FLAG M2 affinity gel (A2220, Sigma), and Alexa Fluor 488-conjugated goat anti-mouse IgG (A-11001, ThermoFisher). To monitor YRDC subcellular localization using the anti-FLAG-tag antibody, mitochondrial fractions were carefully washed with 2 mg/mL digitonin (Sigma) to remove the outer membrane with cytoplasmic components.

### Northern blotting

Total RNA (2 μg) from cultured cells was dissolved by 10% denaturing PAGE, stained with SYBR Gold (Invitrogen), and blotted onto a nylon membrane (Amersham Hybond N+; GE Healthcare) in 1× TBE using a Transblot Turbo apparatus (Bio-Rad). The membrane was air-dried and irradiated twice with UV light (254 nm, 120 mJ/cm^2^; CL-1000, UVP) to crosslink the blotted RNA. Hybridization was performed essentially according to the manufacturer’s instructions (PerfectHyb, TOYOBO) at 52 °C with 4 pmol of 5′-^32^P-labeled oligonucleotides (Supplementary Data 1) specific for each mt-tRNA. The membrane was washed three times with 1× SSC, dried, and exposed to an imaging plate (BAS-MS2040, Fujifilm). Radioactivity was visualized using an FLA-7000 imaging analyzer (Fujifilm).

### Preparation of mt-tRNA transcripts

A series of tRNA mutants were synthesized by in vitro transcription using T7 RNA polymerase^67^. DNA templates for in vitro transcription were constructed by PCR using a series of oligo DNAs (listed in Supplementary Data 1). All mt-tRNA transcripts were gel-purified.

### In vitro reconstitution of t^6^A37

In vitro reconstitution of t^6^A37 on tRNA was carried out at 37 °C for 1 h in a 20-μL reaction mixture containing 100 mM Tricine-NaOH (pH 8.0), 2 mM DTT, 15 mM MgCl_2_, 1 mM ATP, 5 mM *L*-Thr, 40 mM NaHCO_3_, 1 μM mt-tRNA, 1 μM YRDC (17–279a.a.), and 2 μM OSGEPL1 (35–414a.a.). The mt-tRNAs were extracted with phenol/chloroform/isoamyl alcohol (25:24:1, pH 5.2) (Nacalai Tesque), dialyzed, digested with RNase T_1_, and subjected to LC/MS analyses to detect and quantify t^6^A37 formation.

For kinetic analyses, initial velocity of t^6^A37 formation was measured at 37 °C in a 10-μL reaction mixture (for each data point) containing 100 mM Tricine-NaOH (pH 8.0), 2 mM DTT, 15 mM MgCl_2_, 18.75–150 μM ATP, 14.275–114.2 μM [^14^C] *L*-Thr, 5–40 mM NaHCO_3_, 0.4–3.2 μM mt-tRNA, 1 μM YRDC (17–279a.a.), and 2 μM OSGEPL1 (35–414a.a.). To stop the reaction, the mixture was spotted on a Whatman 3MM filter paper, soaked in 5% TCA, washed three times with 5% TCA, and rinsed with ice-cold ethanol. Radioactivity of [^14^C] *L*-Thr on the dried paper was measured by liquid scintillation counting on a Tri-Carb 2810TR (PerkinElmer).

### Kinetic analysis of TC-AMP formation

TC-AMP is an unstable compound with a short half-life^32^. To measure kinetic parameters of TC-AMP formation catalyzed by YRDC, we converted TC-AMP to *N*-carboxythreonyl anhydride (Thr-NCA) under mild alkaline conditions (pH 8.2). Thr-NCA is stable enough to be detected and quantified by thin layer chromatography (TLC). Thr-NCA formation was carried out at 37 °C in a 10-μL reaction mixture (for each data point) containing 50 mM Tricine-NaOH (pH 8.5), 10 mM MgCl_2_, 1 mM ATP, 100 mM NaHCO_3_, and 2 mM [^14^C] *L*-Thr. For kinetic analyses, the concentration of each substrate was altered to 0.1–0.8 mM ATP, 10–80 mM NaHCO_3_, and 50–400 μM *L*-Thr, respectively. To stop the reaction, 0.1 volume of acetic acid was mixed with the solution for each period of time. Aliquots were spotted on silica gel TLC plate (Merck) which was then developed by a solvent consisted of *n*-butanol:acetic acid:water = 4:1:1. Radioactivity of [^14^C] Thr-NCA on the TLC plate was visualized using an imaging plate (BAS-MS2040, Fujifilm) on a fluorimager (FLA-7000, Fujifilm).

### Mitochondrial activities

For mitochondrial isolation, WT or *OSGEPL1* KO HEK293T cells (5 × 10^7^) were homogenized in 3 mL of low buffer (80 mM sucrose, MOPS-NaOH (pH 7.2)) and mixed with equal volume of high buffer (250 mM sucrose, 10 mM MOPS-NaOH (pH 7.2)), followed by centrifugation at 600 *g* for 15 min. The supernatant was collected, homogenized again, and centrifuged at 10,000×*g* for 20 min to collect the mitochondrial fraction as a pellet. The resultant mitochondrial fraction was used to measure complex activities as described^68^. For Complex I, a reaction mixture (1 mL) consisting of 40 μg/mL mitochondria, 50 mM KH_2_PO_4_-KOH (pH 7.4), 1 mM KCN, 50 μM CoQ1, and 75 μM NADH was incubated at 32 °C, and absorbance at 340 nm was monitored by UV-visible spectrophotometer V-630 equipped with a temperature control unit PAC-743R (JASCO). As a negative control, the reaction mixture containing 20 μM rotenone was used. For Complex II, a reaction mixture (1 mL) consisting of 40 µg/mL mitochondria, 50 mM KH_2_PO_4_-KOH (pH 7.4), 20 mM succinate (pH7.2), 5 µg/mL antimycin, 50 µM rotenone, 2 mM KCN, 50 µM 2,6-dichlorophenolindophenol (DCPIP), and 50 µM decylubiquinone (DB) was incubated at 32 °C, and absorbance at 600 nm was monitored. For measurement of Complex III, reduced DB was prepared as follows: 50 µL of 10 mM DB, 5 µL of 0.1 M HCl, and a small piece of potassium borohydride were mixed and incubated at room temperature. Followed by centrifugation, the supernatant was mixed with 5 μL of 1 M HCl. Complex III activity was monitored by absorbance at 550 nm at 32 °C in 1 mL reaction mixture consisting of 40 µg/mL mitochondria, 6 µL of reduced DB, 50 µM cytochrome C (CytoC), 10 mM KH_2_PO_4_-KOH (pH 7.4), 2 mM EDTA, 4 µM rotenone, and 2 mM KCN. For measurement of Complex IV, reduced CytC was prepared as follows: 1 mL of 2.7 mg/mL CytoC and 50 µL of 0.1 M DTT were mixed and reacted at room temperature for 15 min. Complex IV activity was monitored by absorbance at 550 nm at 32 °C in 1 mL reaction mixture consisting of 40 µg/mL mitochondria, 10 mM KH_2_PO_4_-KOH (pH 7.4), and 129 µg/mL reduced CytoC.

To measure the ATP levels, WT or *OSGEPL1* KO HEK293T cells were seeded in 96-well optical-bottom plates (10^4^ cells/well). After 20 h incubation at 37 °C in 5% CO_2_, total ATP in each well was measured using luciferin-luciferase assay solution (CA100, TOYO INK) to calculate the relative ATP levels. Luminescence was detected using a GLOMAX96 Microplate Luminometer (Promega).

Oxygen consumption rates (OCRs) of WT or *OSGEPL1* KO HEK293T cells were measured using an XFp extracellular flux analyzer (Seahorse Bioscience) as described previously^52^. WT and *OSGEPL1* KO cells were seeded in 3 wells each (3 × 10^5^ cells/well) of an XF24 cell culture miniplate, which is manually coated with collagen, and cultured for 24 h and changed the medium (25 mM glucose, 1.25 mM pyruvic acid, pH 7.4 by NaOH). OCR of each well was measured without any inhibitors.

### Pulse labeling of mitochondrial translation

The pulse labeling of mitochondrial protein synthesis was performed essentially as described^69^. WT or *OSGEPL1* KO HEK293T cells (2 × 10^6^ cells) were cultured at 37 °C for 15 min in 6 mL of *L*-glutamine/*L*-cysteine-free DMEM (21013-024, Gibco) containing 10% dialyzed FBS (Gibco), 2 mM *L*-glutamine (Sigma), and 1 mM sodium pyruvate (Gibco), followed by addition of 77 μg/mL emetine (Sigma) and incubation for 5 min. Then, Easy Tag Express Protein Labeling Mix [^35^S] (NEG772007MC, Perkin Elmer) was added and the sample incubated at 37 °C for 1 h to specifically label mitochondrial proteins. Cell lysates (100 μg total proteins) were resolved by Tricine-SDS-PAGE (16.5%) and the gel was CBB-stained and dried on a gel drier (AE-3750 RapiDry, ATTO). Radiolabeled mitochondrial protein products were visualized by exposure to an imaging plate (BAS-MS2040, Fujifilm) on a fluorimager (FLA-7000, Fujifilm).

### Tumor xenograft

HT-29 cells (5 × 10^6^ cells) were transplanted subdermally into BALB/c nude mice (female, 6 week old). Three weeks after the transplantation, the tumors were removed and subjected to RNA extraction. All the animal protocols were approved by the safety division of RIKEN.

### Case report and clinical course for the A15923G mutation

The patient was a female and aged 15 years and 1 month at the time of sample collection. At age 6, she was diagnosed with bilateral hearing impairment, which became progressively worse from around the age of 8 years. She started to use hearing aids at age 9 and underwent cochlear implant surgery at the age of 10 years and 11 months. At around age 10, short stature was noted and a detailed examination was conducted. MRI and CT scans of the head revealed calcification of the bilateral dentate nuclei, caudate nuclei, putamina, globi pallidi, and thalami. She also had elevated levels of lactic acid/pyruvic acid in the blood (23.6/0.87 mg/dL) and spinal fluid (35.0/1.29 mg/dL), pigmentary degeneration of the retina, and proteinuria; consequently, mitochondrial disease was suspected. At around age 12, the patient started to have myoclonic seizures and generalized seizures triggered by visual stimuli such as light. The electroencephalogram (EEG) revealed spike-and-wave discharges (approximately 3 Hz), and treatment with anti-seizure medication was initiated. The clinical course and symptoms were suggestive of MERRF. Occasional headaches were also observed. At the age of 12 years and 10 months, she underwent a muscle biopsy and the muscle pathology revealed scattered cytochrome-*c*-oxidase-negative ragged-red fibers. Full mitochondrial DNA sequencing detected essentially homoplasmic m.15923A >G mutation in muscle. Based on these findings, the diagnosis of mitochondrial disease was established. This study was approved by the IRB of National Center of Neurology and Psychiatry.

### Data availability

The data supporting the findings in this study are available from the corresponding author upon reasonable request.

## Electronic supplementary material

Supplementary Information

Description of Additional Supplementary Files

Supplementary Data 1
