# Supplementary material for: CO2-sensitive tRNA modification associated with human mitochondrial disease
Source: Nat Commun. 2018 May 14;9:1875. doi: 10.1038/s41467-018-04250-4 (PMC5951830; doi:10.1038/s41467-018-04250-4)
Supplement: Supplementary file 1 — Supplementary Information [file 41467_2018_4250_MOESM1_ESM.pdf]

Supplementary information

CO_2-sensitive tRNA modification associated with human mitochondrial disease

Huan Lin[1], Kenjyo Miyauchi[1], Tai Harada[1], Ryo Okita[1], Eri Takeshita[3,4], Hirofumi Komaki[3,4], Kaoru Fujioka[5], Hideki Yagasaki[5], Yu-ichi Goto[2,3,4], Kaori Yanaka[6], Shinichi Nakagawa[6,7], Yuriko Sakaguchi[1], and Tsutomu Suzuki[1],*

1) Department of Chemistry and Biotechnology, Graduate School of Engineering, University of Tokyo, 7-3-1 Hongo, Bunkyo-ku, Tokyo 113–8656, Japan
2) Medical Genome Center, 3) Department of Mental Retardation and Birth Defect Research, National Institute of Neuroscience, 4) Department of Child Neurology, National Center Hospital, National Center of Neurology and Psychiatry, Kodaira, Tokyo, Japan
5) Department of Pediatrics, Interdisciplinary Graduate School of Medicine and Engineering, University of Yamanashi, Japan
6) RNA Biology Laboratory, RIKEN Advanced Research Institute, 2-1 Hirosawa, Wako, Saitama 351-0198, Japan
7) RNA Biology Laboratory, Faculty of Pharmaceutical Sciences, Hokkaido University, Kita 12-jo Nishi 6-chome, Kita-ku, Sapporo 060-0812, Japan

*To whom correspondence should be addressed.
Tel: +81-3-5841-8752
E-mail: ts@chembio.t.u-tokyo.ac.jp

tRNA	sequence	modification	*m/z*		
**mt-tRNA^{Asn}**	**UUAACUAAGp**		z=-2	z=-3	z=-4
(RNase T_1)		A37	1450.18	966.45	724.59
		t^6A37	1522.70	1014.80	760.85
**mt-tRNA$^{Ser(AGY)}$**	**CUAACUCAUGp**		z=-3	z=-4	z=-5
(RNase T_1)		A37	1060.13	794.85	635.68
		t^6A37	1108.48	831.11	664.68
**mt-tRNA^{Ile}**	**AUAGp**		z=-1	z=-2	
(RNase T_1)		A37	1326.18	662.59	
		t^6A37	1471.22	735.11	
**mt-tRNA^{Thr}**	**UAAACCGp**		z=-2	z=-3	
(RNase T_1)		A37	1132.15	754.43	
		t^6A37	1204.67	802.78	
**mt-tRNA^{Thr}**	**AGACp**		z=-1	z=-2	
(RNase A)		A37	1325.20	662.09	
		t^6A37	1470.23	734.61	
**mt-tRNA^{Thr}**	**UCUUGp**		z=-1	z=-2	
(RNase T_1)		C32	1585.17	792.08	
		m^3C32	1599.18	799.09	
**mt-tRNA^{Lys}**	**CAUUAACCUUUUAAGp**	A37, U34	z=-6	z=-7	
(RNase T_1)		A, U	792.26	678.93	
		A, s^2U	794.92	681.22	
		A, τm^5U	815.09	698.51	
		A, τm^5s^2U	817.76	700.79	
		t^6A, U	816.43	699.65	
		t^6A, s^2U	819.09	701.94	
		t^6A, τm^5U	839.27	719.23	
		t^6A, τm^5s^2U	841.93	721.51	
**ct-tRNA^{Ile}**	**AUAACGp**		z=-2	z=-3	
(RNase T_1)		A37	979.63	652.75	
		t^6A37	1052.15	701.10	

Supplementary Table 1. List of *m/z* values of RNA fragments for calculating modification frequencies.

The residues with modification are colored in red. An RNase used for digesting tRNA is shown in parenthesis.

Substrates	K_m	k_{cat} ($\times 10^{-3}$, s^{-1})	k_{cat}/K_m ($s^{-1} \cdot mM^{-1}$)
mt-tRNA^{Thr}	0.42 ± 0.05 μM	0.95 ± 0.028	2.26
ATP	76 ± 10 μM	0.87 ± 0.055	0.011
L-threonine	39 ± 3.7 μM	1.08 ± 0.042	0.028
HCO_3^-	31 ± 3.8 mM	1.54 ± 0.10	0.000050

Supplementary Table 2. Kinetic parameters of mitochondrial t^6A37 formation

Substrates	K_m	k_{cat} ($\times 10^{-2}$, s^{-1})
ATP	170 ± 220 μM	3.9 ± 2.23
L-threonine	190 ± 60 μM	2.7 ± 0.29
HCO_3^-	13 ± 3.8 mM	2.5 ± 0.22

Supplementary Table 3. Kinetic parameters of NCA formation by YRDC

mt-tRNAs (codons)		Asn (AAY)	Ile (AUY)	Lys (AAR)	Ser (AGY)	Thr (ACN)	Total usage (%)
I	ND1	4.1	7.2	2.2	0.9	11	25.4
	ND2	5.8	8.9	3.5	1.4	12.4	32.0
	ND3	3.5	7.8	2.6	0.9	6.1	20.9
	ND4	5.0	8.5	2.4	2.2	10.5	28.6
	ND4L	6.1	7.1	0	0	5.1	18.3
	ND5	5.5	8.9	3.5	15.7	10.8	44.4
	ND6	2.3	6.9	1.1	2.9	1.7	14.9
III	Cytb	3.9	10.3	2.4	1.1	7.9	25.6
IV	CO I	3.3	7.4	1.9	0.8	6.6	20.0
	CO II	3.1	9.7	1.8	0.4	9.3	24.3
	CO III	2.3	5.4	1.1	1.5	9.2	19.5
V	ATPase6	4.8	12.8	2.6	1.3	11	32.5
	ATPase8	7.2	4.3	10.1	0	11.6	33.2

Supplementary Table 4. Usage of codons read by tRNAs bearing t^6A37 in human mitochondria

Usage (%) of codons read by five mt-tRNAs bearing t^6A37 in all 13 proteins encoded by human mtDNA.

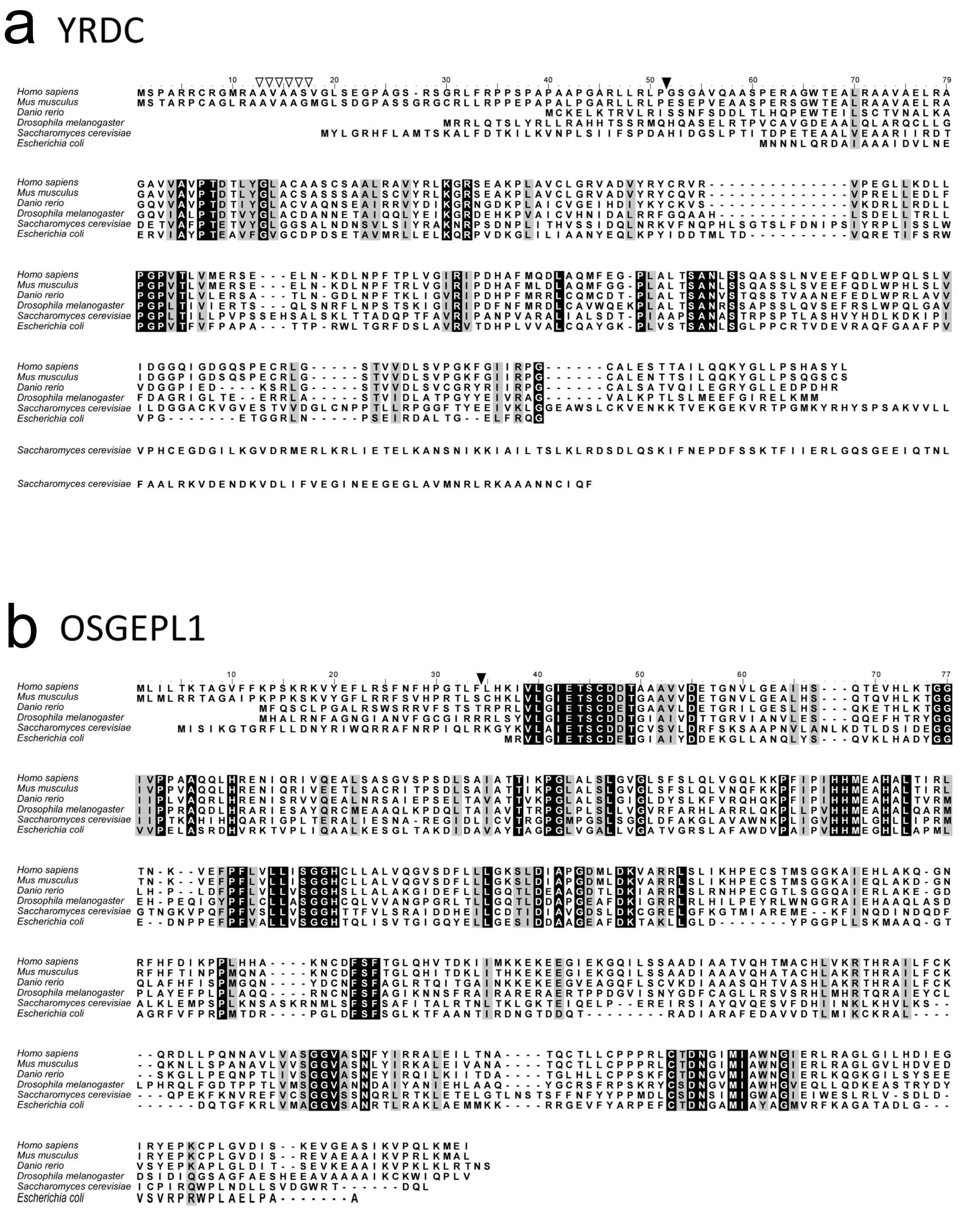

Supplementary Figure 1. Sequence alignment of YRDC and OSGEPL1.

(a) Sequence alignment of YRDC homologs from *Homo sapiens* (NP_078916.3), *Mus musculus* (NP_705794.2), *Danio rerio* (NP_001077336.1), *Drosophila melanogaster* (NP_001027444.1), *Saccharomyces cerevisiae* (NP_011346.1), and *Escherichia coli*

(NP_417741.1). Black and grey boxes represent identical and similar amino acids, respectively. Multiple cleavage sites within the long and the short isoforms of YRDC expressed in HeLa cells are indicated by white and black arrowheads, respectively.
(b) Sequence alignment of OSGEPL1 homologs in *Homo sapiens* (NP_071748.2), *Mus musculus* (NP_001272768.1), *Danio rerio* (NP_001005301.1), *Drosophila melanogaster* (NP_001245765.1), *Saccharomyces cerevisiae* (NP_010179.1), and *Escherichia coli* (NP_417536.1). Black and grey boxes represent identical and similar amino acids, respectively. The cleavage site in the MTS of OSGEPL1 expressed in HeLa cells is indicated by a black arrowhead.

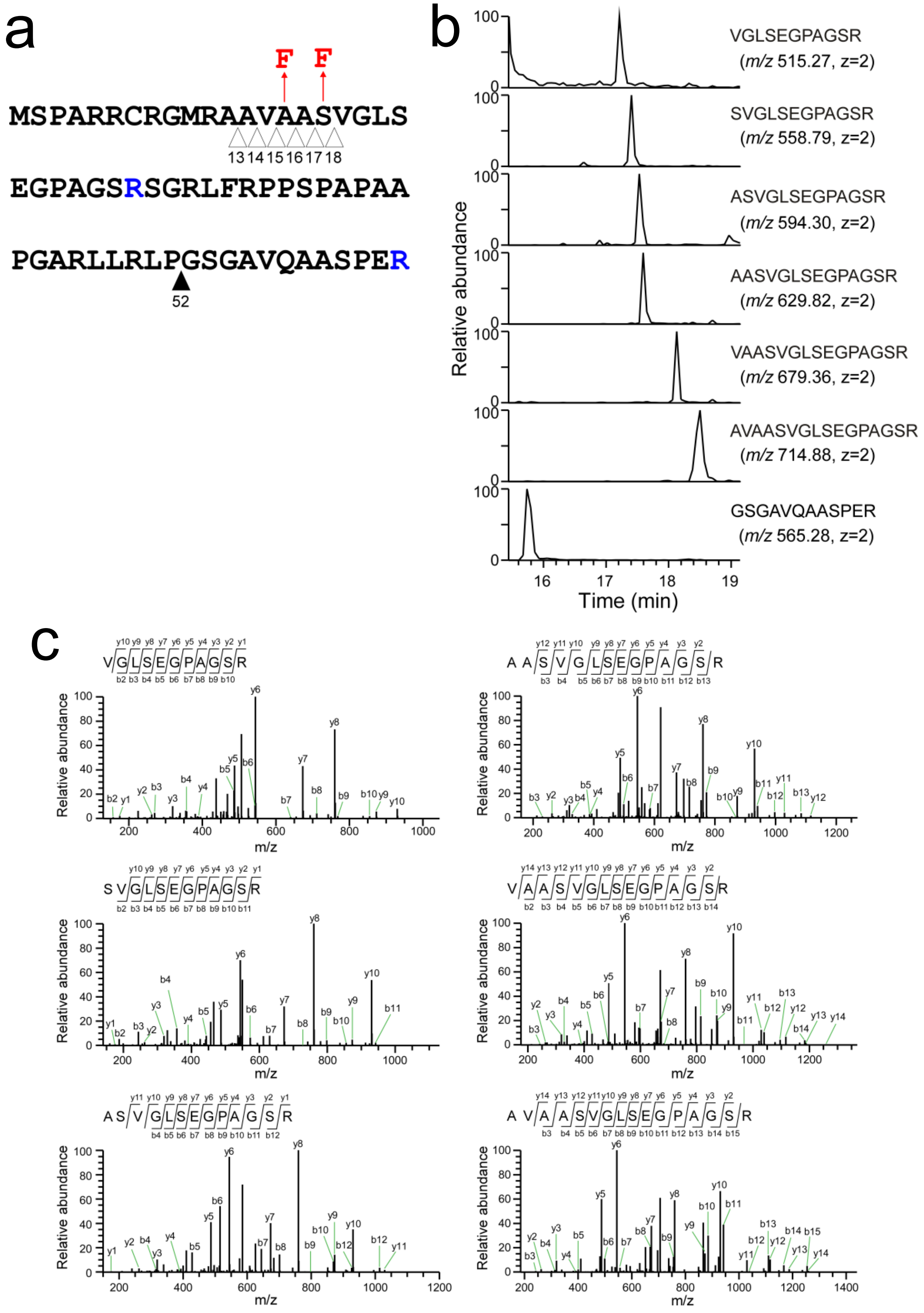

Supplementary Figure 2. Multiple cleavage sites in the MTS of YRDC-FLAG expressed in HeLa cells.

(a) Peptide sequence of the MTS of YRDC with multiple cleavage sites in the long (white arrowheads) and the short (black arrowhead) isoforms. Point mutations A15F and S17F are indicated.
(b) Mass chromatograms of N-terminal tryptic peptides of YRDC. Divalent cations of proton-adducts of N-terminal peptides starting from positions 13–18 and 52 (from top to bottom panels) are indicated.
(c) CID spectra of each N-terminal tryptic peptide derived from the long isoforms of YRDC. Product ions are assigned on the peptide sequence.

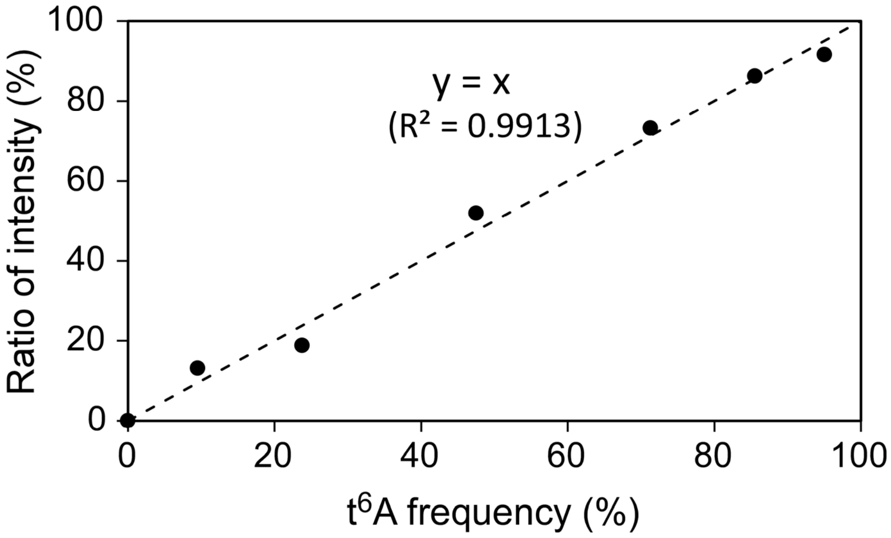

Supplementary Figure 3. ESI detection efficiency of RNA fragments with varying t^6A frequency.

Native human mt-tRNA^{Ile} isolated from WT HEK293T cells and its unmodified one isolated from *OSGEPL1* KO cells (KO#2) were mixed with different mixing ratios, digested by RNase T_1 and subjected to LC/MS. The peak area ratio of the A37-containing fragments with and without t^6A was linearly regressed on the mixing ratio (slope=0.95, r^2=0.9913). Actual t^6A frequency of the native mt-tRNA^{Ile} was calculated to be 95% from the slope. Then, the mixing ratios were transformed to t^6A frequencies on the x-axis to generate a plot of intensity ratio (%) against t^6A frequency (%) (slope=1, r^2=0.9913).

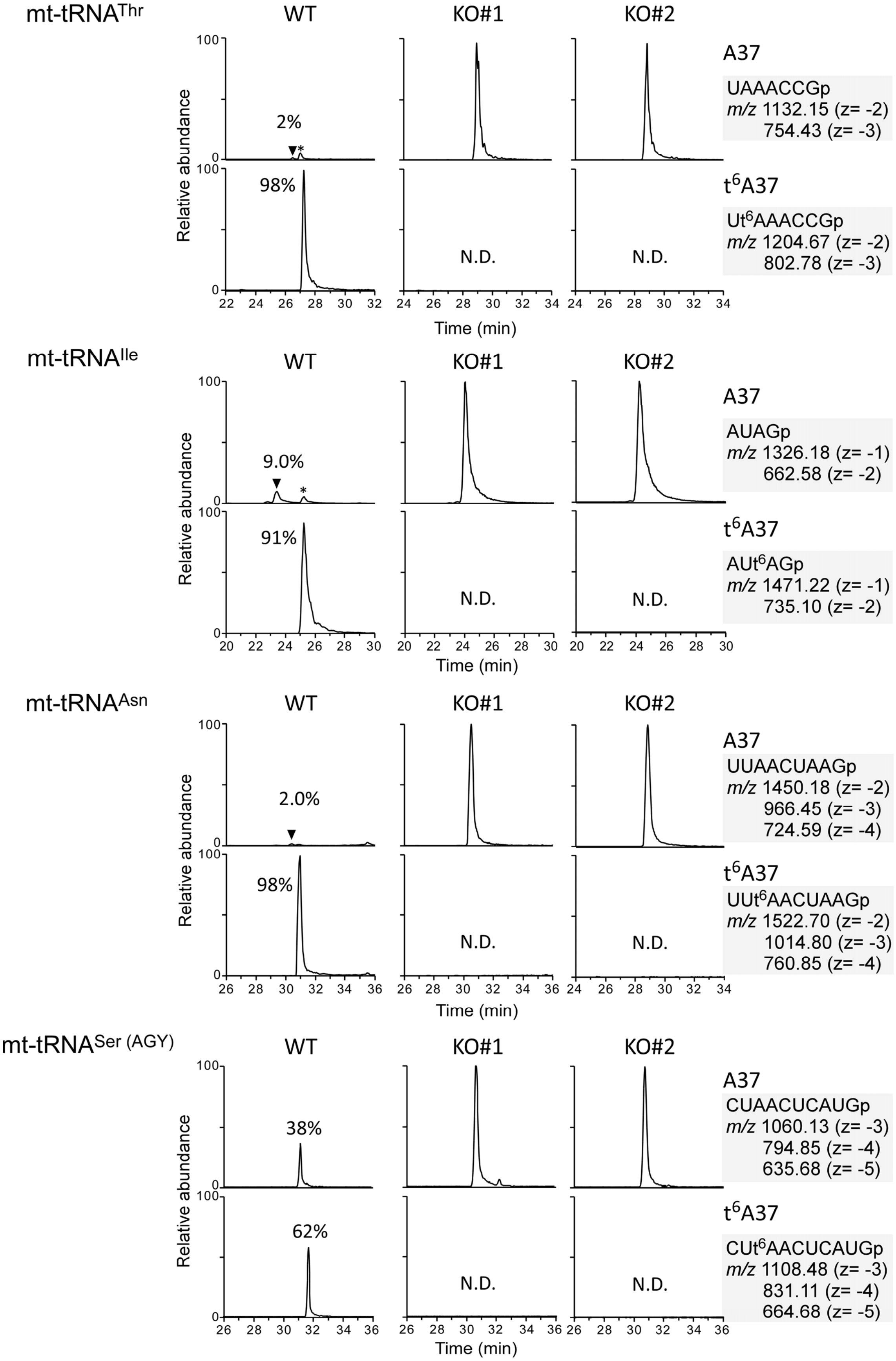
mt-tRNA^{Thr}
WT
KO#1
KO#2
A37
UAAACCGp
m/z 1132.15 (z= -2)
754.43 (z= -3)
2%
98%
t^{6}A37
Ut^{6}AAACCGp
m/z 1204.67 (z= -2)
802.78 (z= -3)
N.D.
N.D.
Relative abundance
Time (min)
mt-tRNA^{Ile}
WT
KO#1
KO#2
A37
AUAGp
m/z 1326.18 (z= -1)
662.58 (z= -2)
9.0%
91%
t^{6}A37
AUt^{6}AGp
m/z 1471.22 (z= -1)
735.10 (z= -2)
N.D.
N.D.
Relative abundance
Time (min)
mt-tRNA^{Asn}
WT
KO#1
KO#2
A37
UUAACUAAGp
m/z 1450.18 (z= -2)
966.45 (z= -3)
724.59 (z= -4)
2.0%
98%
t^{6}A37
UUt^{6}AACUAAGp
m/z 1522.70 (z= -2)
1014.80 (z= -3)
760.85 (z= -4)
N.D.
N.D.
Relative abundance
Time (min)
mt-tRNA$^{Ser\ (AGY)}$
WT
KO#1
KO#2
A37
CUAACUCAUGp
m/z 1060.13 (z= -3)
794.85 (z= -4)
635.68 (z= -5)
38%
62%
t^{6}A37
CUt^{6}AACUCAUGp
m/z 1108.48 (z= -3)
831.11 (z= -4)
664.68 (z= -5)
N.D.
N.D.
Relative abundance
Time (min)

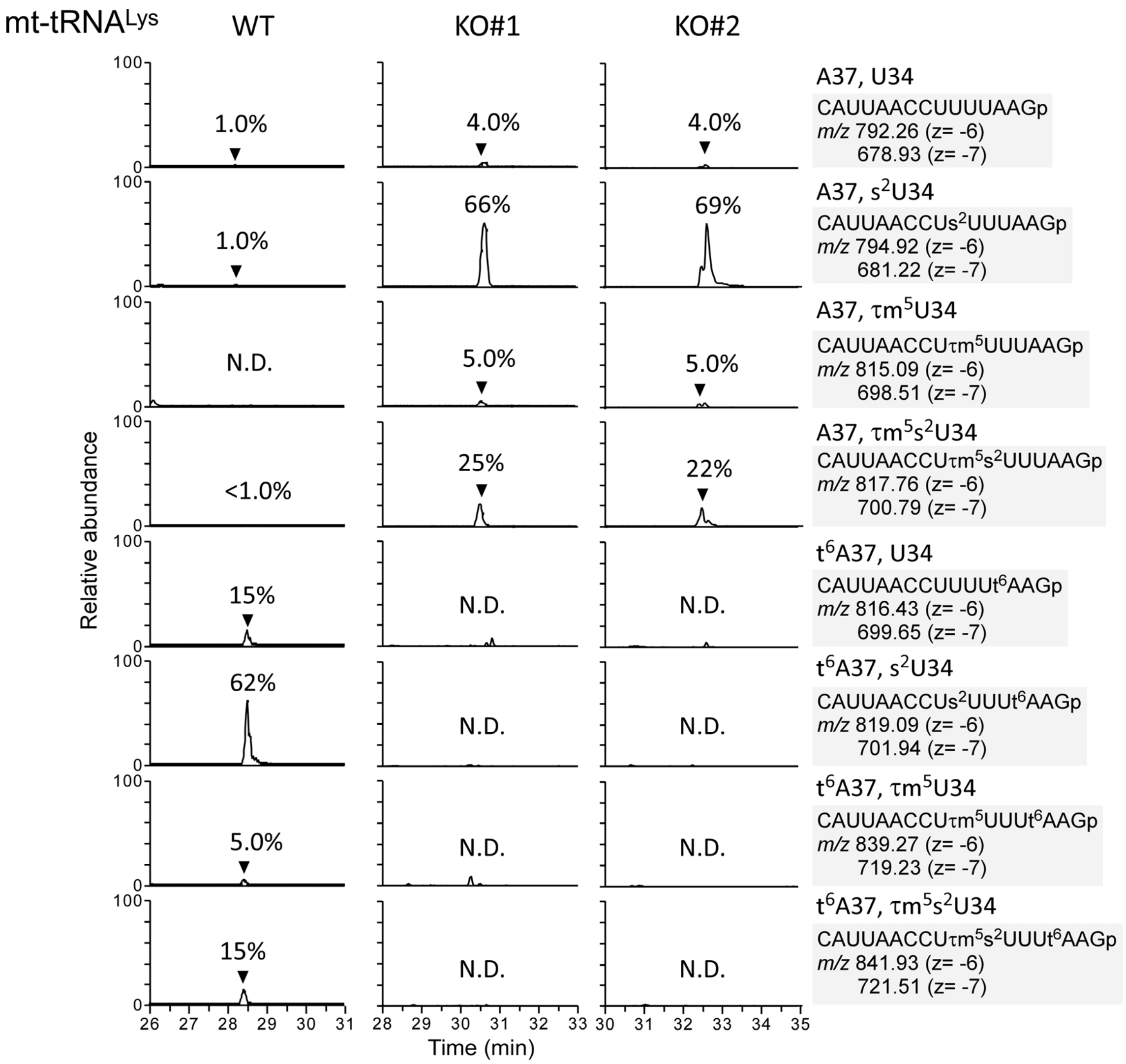

Supplementary Figure 4. Lack of t^{6}A37 in five mt-tRNAs from *OSGEPL1* KO cells. Extracted ion chromatograms (XIC) generated by integration of multiply-charged negative ions of A37-containing fragments of human mt-tRNAs for Thr, Ile, Asn, Ser (AGY) and Lys harboring A37 (upper panels) or t^{6}A37 (lower panels) (Supplementary Table 1) isolated from WT HEK293T (left panels), KO#1 (middle panels), and KO#2 (right panels). N.D., not detected. Intensity fractions (%) of modified or unmodified fragments are indicated.

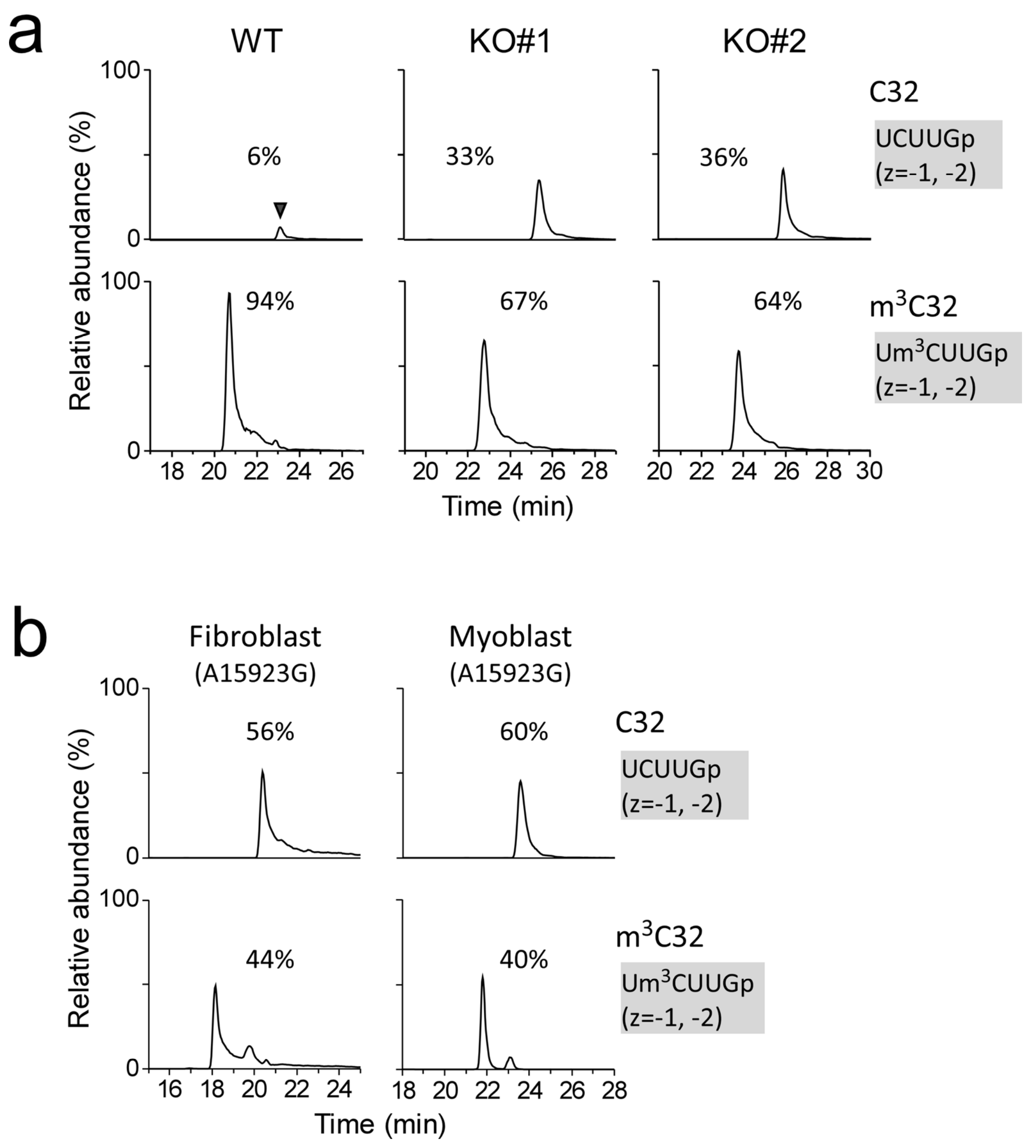

Supplementary Figure 5. Hypomodification of m^{3}C32 in mt-tRNA^{Thr} from *OSGEPL1* KO cells and cells from a patient with MERRF-like symptoms harboring the A15923G mutation.

(a) XICs generated by integration of multiply-charged negative ions of C32-containing fragments of human mt-tRNA^{Thr} bearing C32 (upper panels) and m^{3}C32 (lower panels) (Supplementary Table 1) isolated from WT HEK293T (left panels), *OSGEPL1* KO#1 (middle panels), and *OSGEPL1* KO#2 (right panels). Intensity fractions (%) of modified or unmodified fragments are indicated.

(b) XICs generated by integration of multiply-charged negative ions of C32-containing fragments of human mt-tRNA^{Thr} bearing C32 (upper panels) and m^{3}C32 (lower panels) (Supplementary Table 1) isolated from the fibroblasts (left panels) and myoblasts (right panels) of a patient with the A15923G mutation.

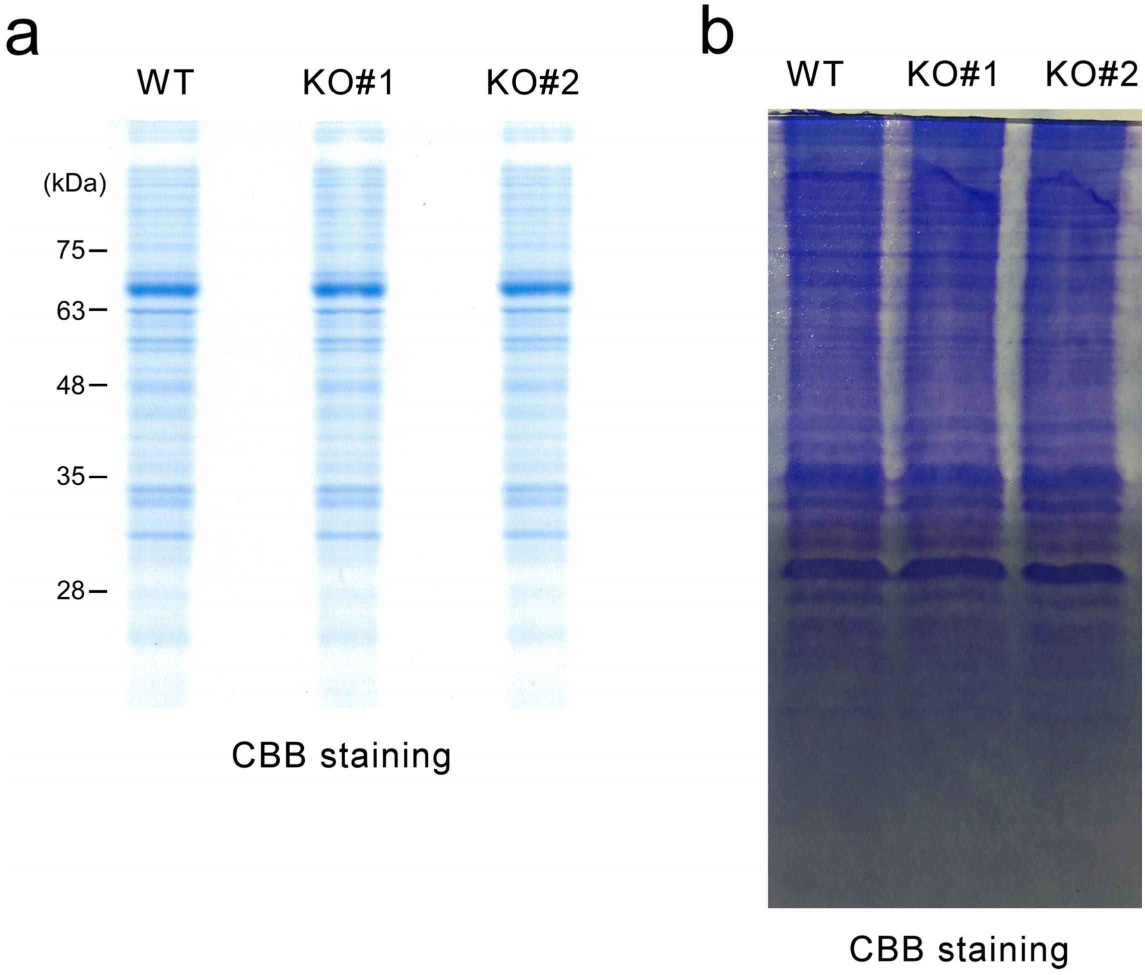

Supplementary Figure 6. Loading controls for protein analyses.

(a) CBB staining of mitochondrial fractions from WT HEK293T, *OSGEPL1* KO#1, and *OSGEPL1* KO#2 used for western blotting (shown in Fig. 4e).

(b) CBB staining of total proteins from WT HEK293T, *OSGEPL1* KO#1, and *OSGEPL1* KO#2 used for the pulse-labeling experiment (shown in Fig. 4f).

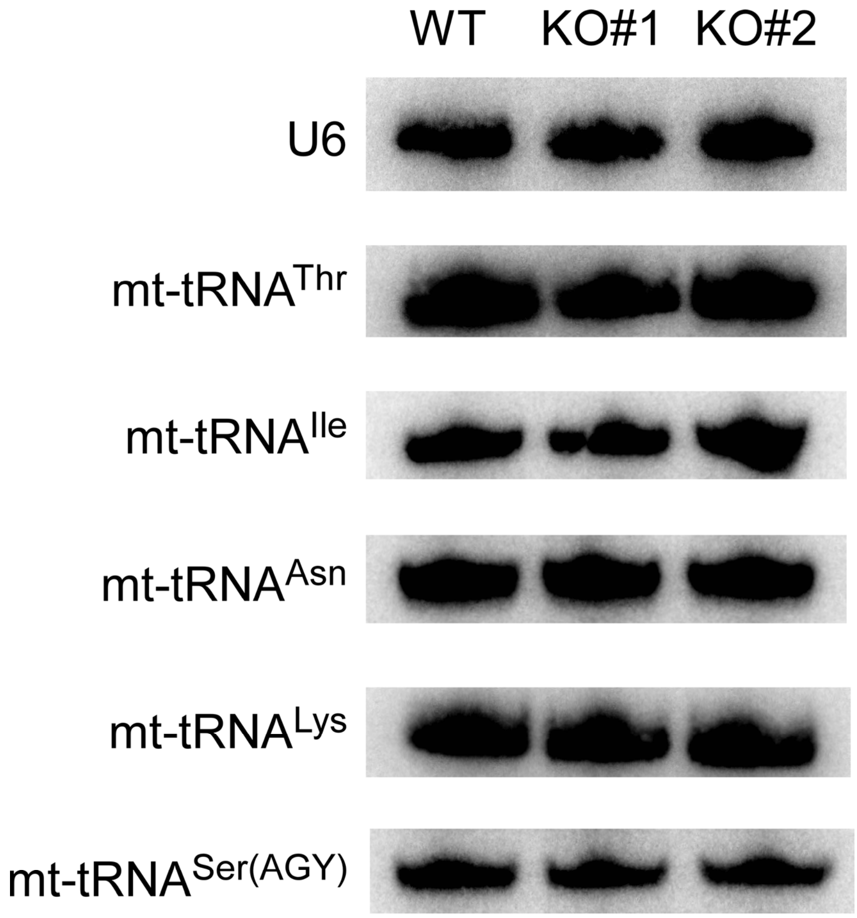

Supplementary Figure 7. Northern blotting of five mt-tRNAs in WT HEK293T and *OSGEPL1* KO cells.

U6 snRNA was detected as a loading control.

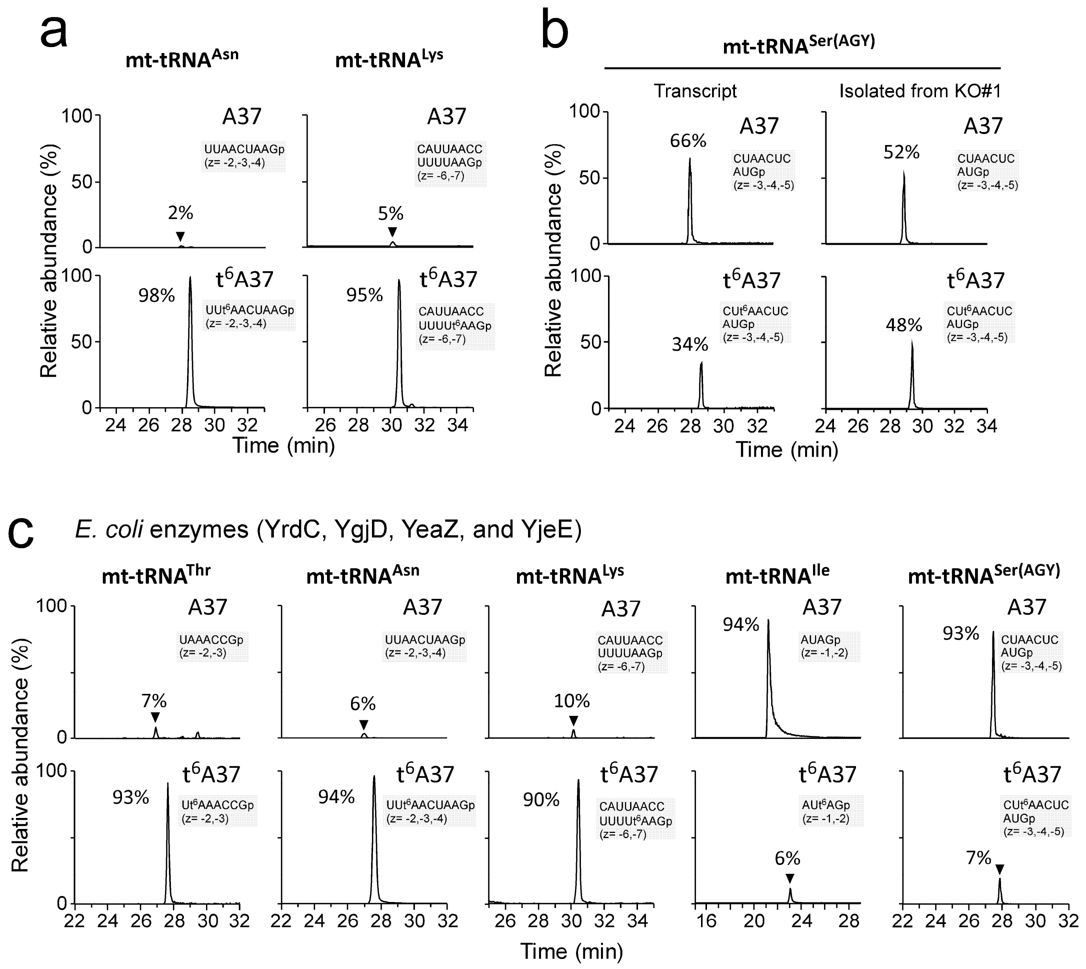

Supplementary Figure 8. *In vitro* reconstitution of t^{6}A37 on mt-tRNAs.

(a) *In vitro* formation of t^{6}A37 on mt-tRNAs for Asn and Lys. XICs generated by integration of multiply-charged negative ions of A37-containing fragments of mt-tRNAs for Asn (left panels) and Lys (right panels) bearing A37 (top) and t^{6}A37 (bottom) (Supplementary Table 1). Intensity fractions (%) of modified or unmodified fragments are indicated.

(b) *In vitro* formation of t^{6}A37 on mt-tRNA$^{Ser(AGY)}$ transcript (left panels) or native mt-tRNA$^{Ser(AGY)}$ isolated from *OSGEPL1* KO#1 cells (right panels). XICs generated by integration of multiply-charged negative ions of A37-containing fragments mt-tRNA$^{Ser(AGY)}$ bearing A37 (top) and t^{6}A37 (bottom) (Supplementary Table 1). Intensity fractions (%) of modified or unmodified fragments are indicated.

(c) *In vitro* formation of t^{6}A37 on five mt-tRNAs mediated by *E. coli* t^{6}A37-modifying enzymes (YrdC, YgjD, YeaZ, and YjeE). XICs generated by integration of multiply-charged negative ions of A37-containing fragments of respective tRNAs bearing A37 (top) and t^{6}A37 (bottom) (Supplementary Table 1). Intensity fractions (%) of modified or unmodified fragments are indicated.

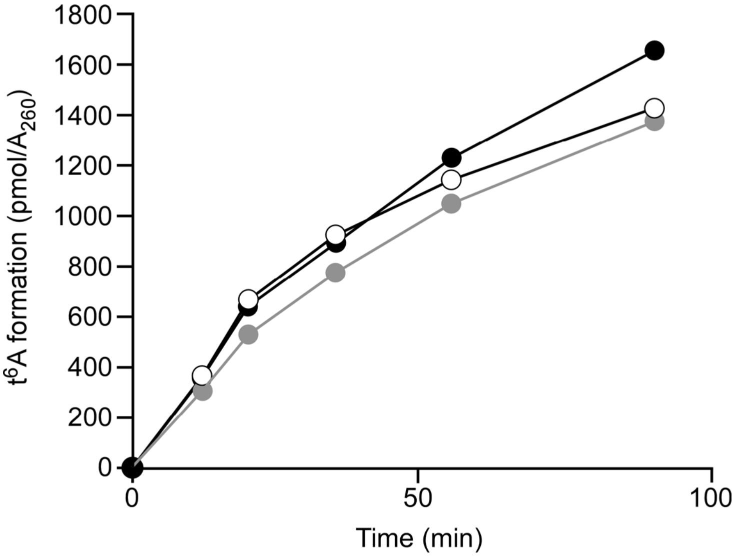

Supplementary Figure 9. t^6A37-forming activity of YRDC isoforms.

In vitro formation of t^6A37 on mt-$tRNA^{Asn}$ with YRDC isoforms with different N-termini: full length (black circle), 17-279 a.a. (gray circle) and 52-279 a.a. (white circle).

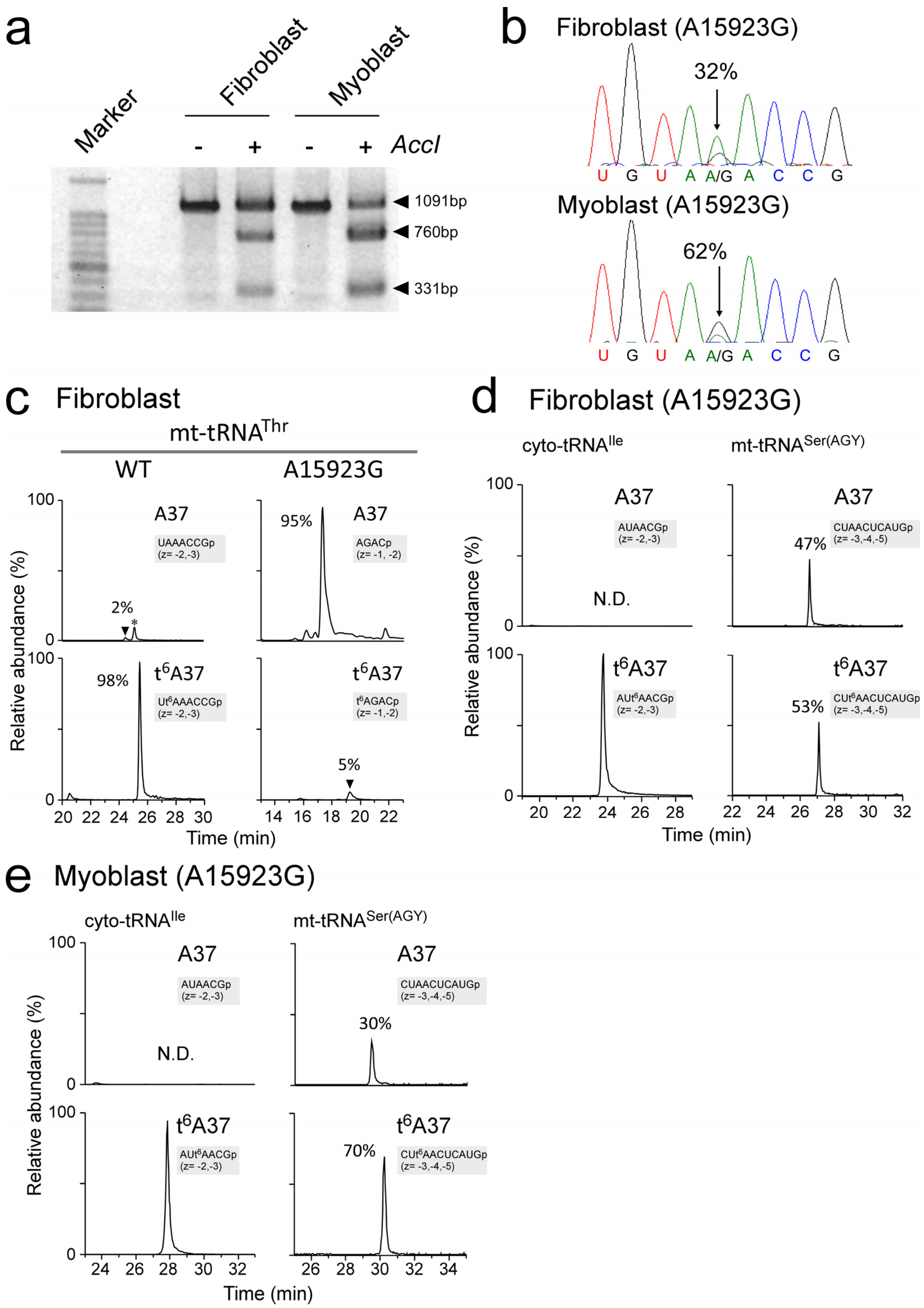

Supplementary Figure 10. Impairment of t^{6}A37 in mt-tRNA^{Thr} with the A15923G mutation isolated from fibroblasts and myoblasts of a patient with MERRF-like symptoms.

(a) Detection of the A15923G mutation in mtDNA by *AccI* digestion. DNA fragments (1091 bp) amplified from mtDNAs from the patient's fibroblasts and myoblasts using a set of specific primers (Supplementary Table 5) were treated with (+) or without (-) *AccI* and the digestion products subjected to agarose gel electrophoresis. Digestion of the 1091 bp DNA containing the A15923G mutation yielded two fragments of 760 bp and 331 bp.

(b) Sequence chromatogram of mtDNAs from the patient's fibroblasts and myoblasts. Mutation rate of position 15923 was calculated from the A-to-G ratio of the peak height.

(c) XICs generated by integration of multiply-charged negative ions of A37-containing fragments of mt-tRNA^{Thr} with A37 (upper panels) and t^{6}A37 (lower panels) (Supplementary Table 1) isolated from WT (left panels) and patient fibroblasts harboring the A15923G mutation (right panels). Intensity fractions (%) of modified or unmodified fragments are indicated.

(d) XICs generated by integration of multiply-charged negative ions of A37-containing fragments of ct-tRNA^{Ile} (left panels) and mt-tRNA$^{Ser(AGY)}$ (right panels) (Supplementary Table 1) isolated from the patient's fibroblasts. Intensity fractions (%) of modified or unmodified fragments are indicated. N.D., not detected.

(e) XICs generated by integration of multiply-charged negative ions of A37-containing fragments of ct-tRNA^{Ile} (left panels) and mt-tRNA$^{Ser(AGY)}$ (right panels) (Supplementary Table 1) isolated from the patient's myoblasts. Intensity fractions (%) of modified or unmodified fragments are indicated. N.D., not detected.

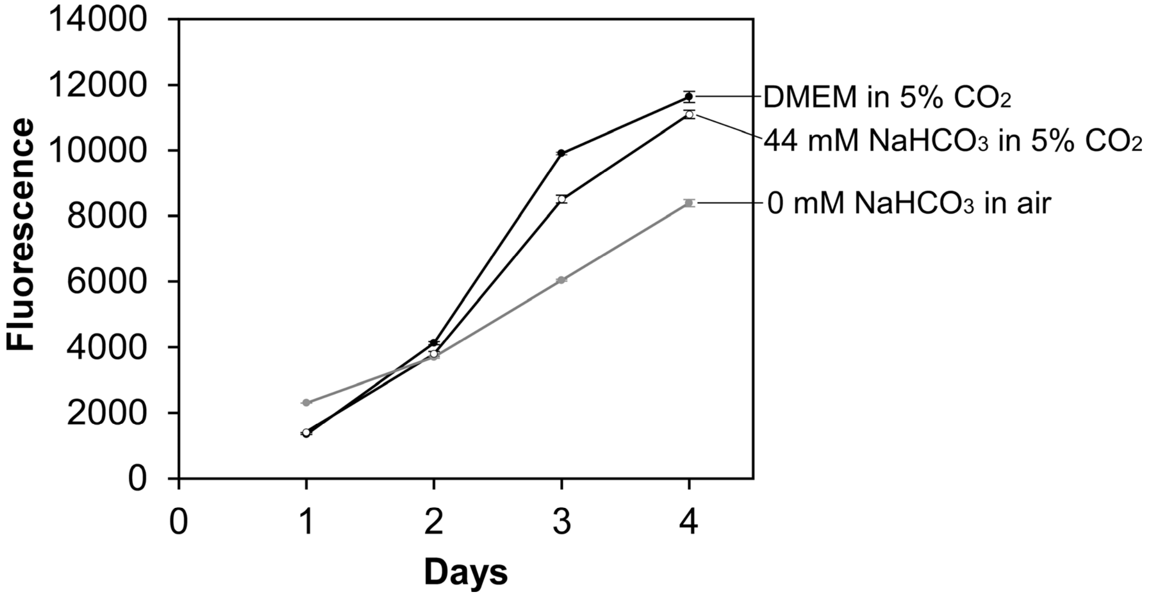

Supplementary Figure 11. Growth curves for WT HEK293T cultured with or without CO_2/HCO_3^-. HEK293T cells were cultured in DMEM medium under 5% CO_2 (black circles), non-bicarbonate DMEM with 44mM $NaHCO_3$ under 5% CO_2 (white circles), or non-bicarbonate DMEM under air (gray circles). Mean values ± s.e.m. of four independent cultures are plotted.

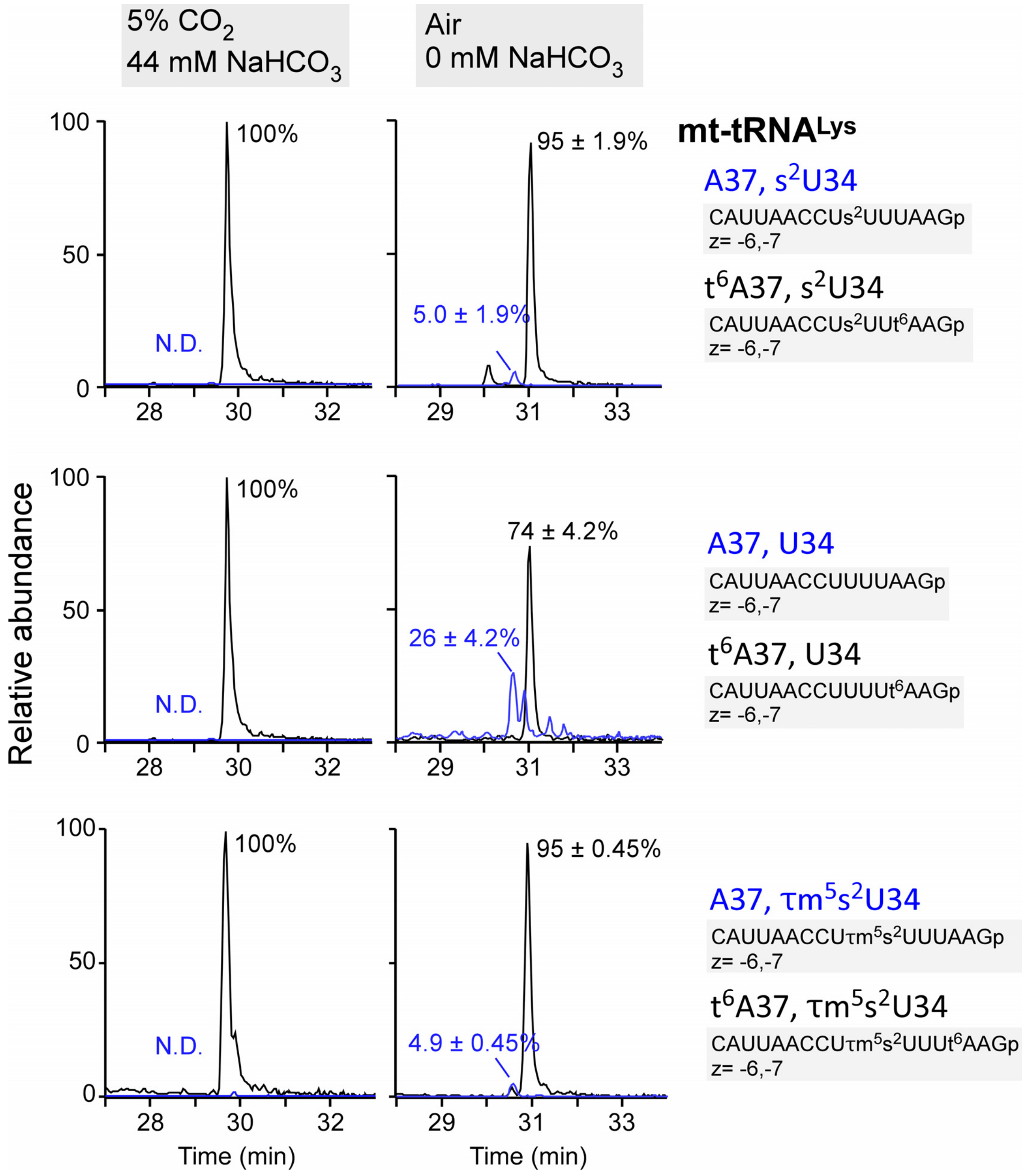

Supplementary Figure 12. Hypomodification of t^6A37 in mt-tRNA^{Lys} with different U34 modifications in HEK293T cells cultured in non-bicarbonate medium.

XICs generated by integration of multiply-charged negative ions of A37-containing fragments of mt-tRNA^{Lys} with s^2U34 (upper panels), unmodified U34 (middle panels) and $\tau m^5 s^2$U34 (lower panels) (Supplementary Table 1) isolated from HEK293T cells cultured with normal DMEM medium (44 mM $NaHCO_3$) in 5% CO_2 (left panels) and non-bicarbonate medium in air (right panels). t^6A frequencies (%) are described as mean values ± s.d. of technical triplicate.

a

b

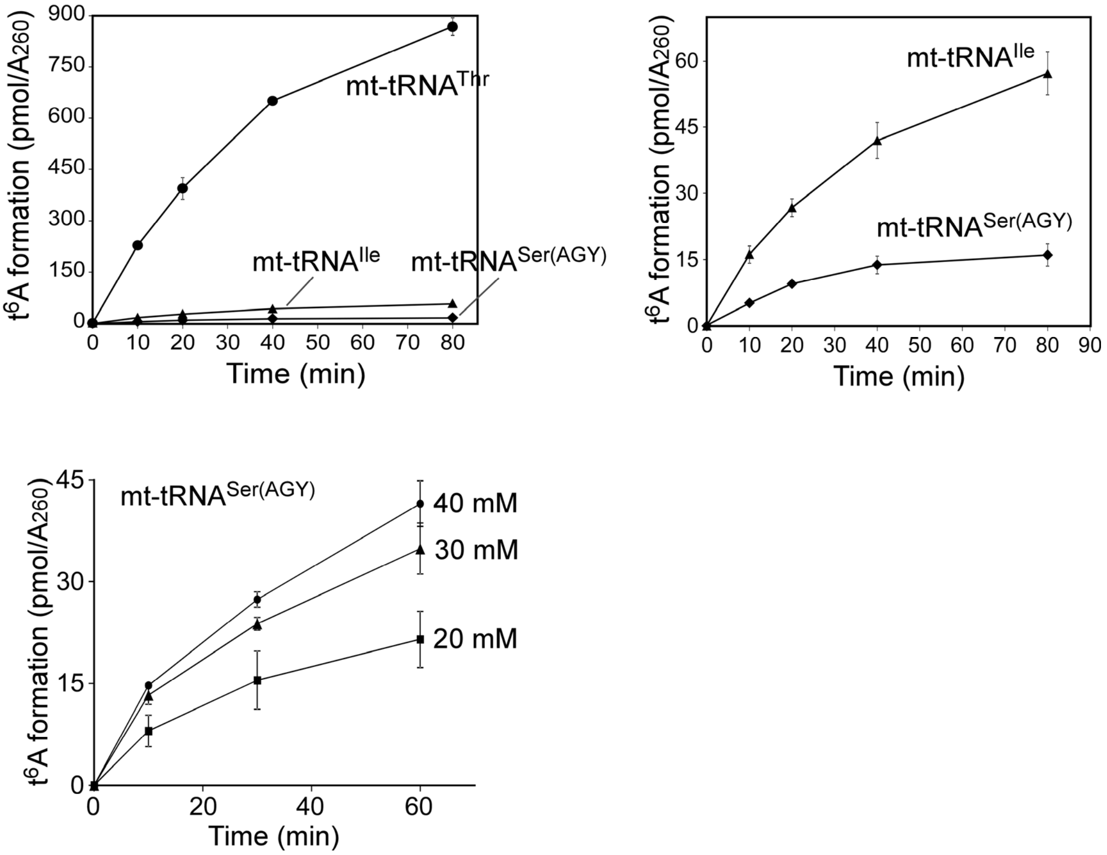

Supplementary Figure 13. Time-course of t^6A37 formation *in vitro* in three mt-tRNAs.

(a) Rates of t^6A37 formation on mt-tRNAs for Ile and Ser(AGY) were much slower than that on mt-tRNA^{Thr}. Right-hand graph is an expanded version of the left-hand graph, intended to emphasize the difference between mt-tRNA$^{Ser(AGY)}$ and mt tRNA^{Ile}. Mean values ± s.d. of biological triplicates are shown.

(b) Bicarbonate-sensitive t^6A37 formation on mt-tRNA$^{Ser(AGY)}$. Time-course of t^6A37 formation *in vitro* in each tRNA was carried out in different concentrations of bicarbonate (20, 30, or 40 mM). Mean values ± s.d. of biological triplicates are shown.

a Solid tumor xenograft

b Culture cell

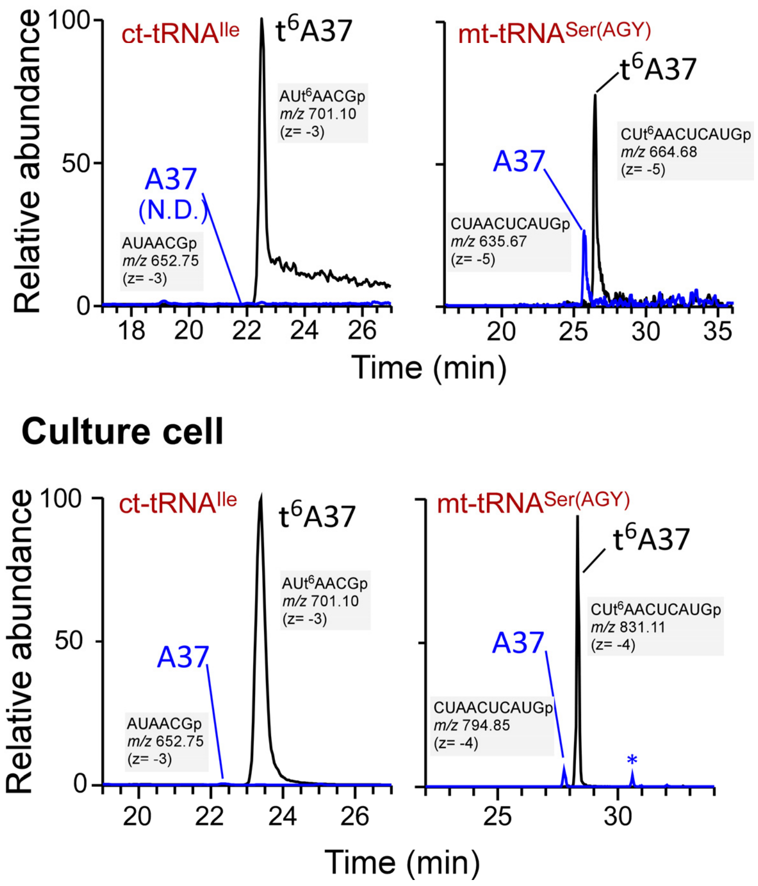

Supplementary Figure 14. t^{6}A status in tRNAs isolated from solid tumors and culture cells of HT29.

Mass chromatograms show A37-containing fragments of ct-tRNA^{Ile} (left panel), and mt-tRNA$^{Ser(AGY)}$ (right panel) bearing A37 (blue) and t^{6}A37 (black), isolated from subdermal HT-29 xenografts (a), and from *in vitro* culture of HT-29 (b).

Fig. 2b

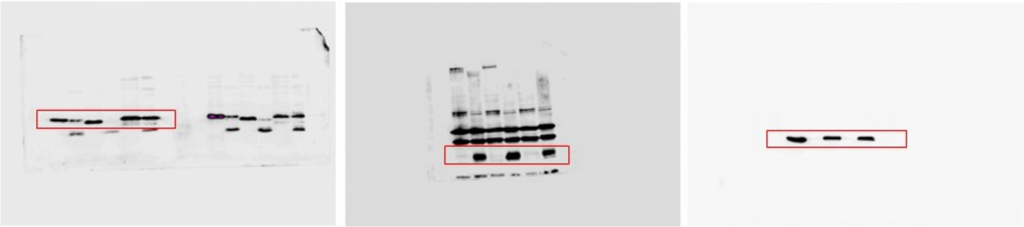

Fig. 3d

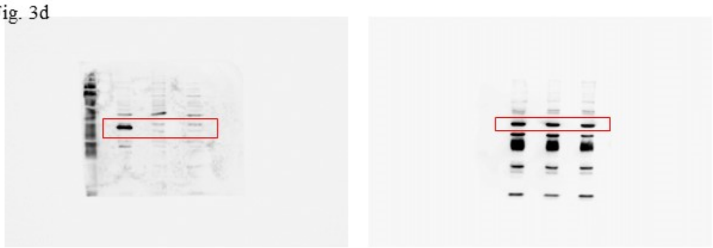

Fig. 4e

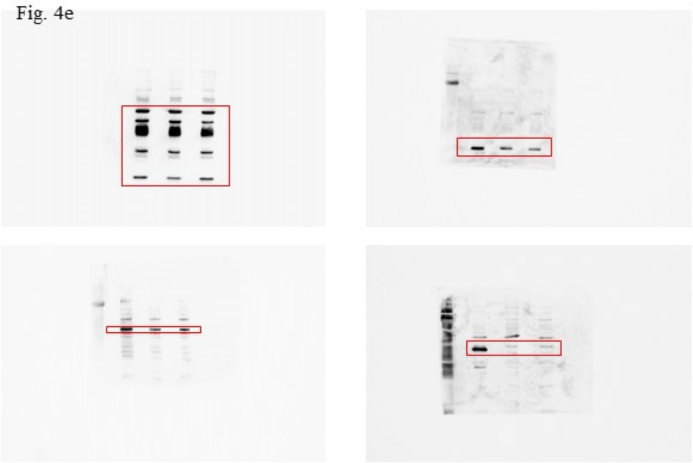

Fig. 4f

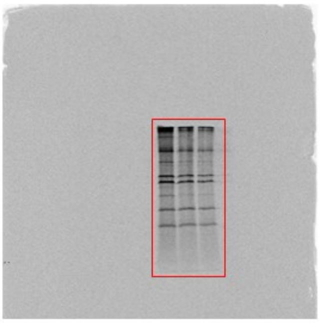

Fig. 4g

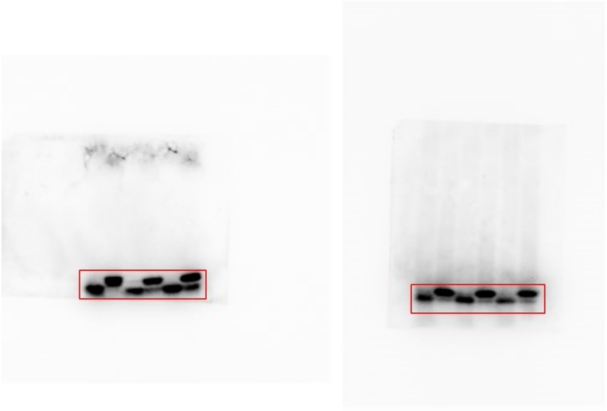

Supplementary Figure 15. Full-size images for the blotting.

The cut region of each image in the indicated figure is red-boxed.
