# Supplementary material for: CO2-sensitive tRNA modification associated with human mitochondrial disease
Source: Nat Commun. 2018 May 14;9:1875. doi: 10.1038/s41467-018-04250-4 (PMC5951830; doi:10.1038/s41467-018-04250-4)
Supplement: Supplementary file 2 — Description of Additional Supplementary Files [file 41467_2018_4250_MOESM2_ESM.pdf]

Description of Additional Supplementary Files

File Name: Supplementary Data 1
Description: List of primers and probes used in this study.
